# The implementation and effectiveness of outlet‐level healthy food and beverage accreditation schemes: A systematic review

**DOI:** 10.1111/obr.13556

**Published:** 2023-02-08

**Authors:** Oliver Huse, Sally Schultz, Tara Boelsen‐Robinson, Jaithri Ananthapavan, Anna Peeters, Gary Sacks, Miranda R. Blake

**Affiliations:** ^1^ Global Centre for Preventive Health and Nutrition (GLOBE), Institute for Health Transformation, Faculty of Health Deakin University Geelong Australia; ^2^ Deakin Health Economics, Institute for Health Transformation, Faculty of Health Deakin University Geelong Australia

**Keywords:** accreditation, food supply, nutrition policy

## Abstract

Healthy food outlet accreditation schemes represent an avenue for incentivizing food retailers to promote healthy eating patterns by improving the healthiness of food environments. This systematic review aimed to (i) assess the impact of food outlet‐level accreditation schemes on outlet practices and customer purchases and (ii) identify barriers and enablers to scheme implementation. Peer‐reviewed and grey literature were systematically searched. Eligible studies related to outlet‐level food and beverage accreditation schemes across any food retail setting. Findings were narratively synthesized by retailer type according to (i) scheme characteristics (governance, targeted products, support, and monitoring); (ii) scheme outcomes (rate of uptake, proportion of certified retailers, impact on purchasing, customer perspectives, and retailer perspectives); and (iii) barriers and enablers to implementation. From 21,943 records screened, 48 were included, covering 26 schemes. Most (18) targeted restaurants or convenience stores. Average uptake was 65% of all outlets approached to participate. Implementation of accreditation schemes was associated with healthier customer purchases in convenience stores, schools, and hospitals, but evidence from restaurants was mixed. Enablers of scheme implementation included support for implementation and maintenance, flexible scheme criteria, and motivated retail staff. Healthy food outlet accreditation schemes represent a promising mechanism for engaging retailers to improve the healthiness of food retail environments.

## 1 BACKGROUND

Unhealthy eating patterns are associated with many adverse health impacts, including overweight and obesity, cancers, diabetes, and heart disease,^1^, ^2^ and are a leading driver of morbidity and premature mortality.^3^ Food retail outlets have been identified as a key driver of the healthiness of population eating patterns^4^, ^5^ and so have been identified as potential venues for obesity prevention.^6^, ^7^ In particular, the consumer nutrition environment (the surroundings, opportunities, and conditions that consumers encounter in a food retail outlet, including the physical, economic, policy, and sociocultural environments) is recognized as a major factor influencing eating patterns.^8^, ^9^ Correspondingly, initiatives within the consumer nutrition environment that seek to improve population eating patterns are potentially powerful from a public health perspective. Multiple systematic reviews have found that interventions that change food and beverage environments so that merchandising and marketing of foods and beverages favors healthy options can lead to healthier purchasing and consumption.^10^, ^11^

Healthy food and beverage interventions have been previously characterized as modifying one or more of the “4Ps” of food environment merchandising: the available “products,” the presence and use of “promotions” to advertise those products, the “prices” at which those products are sold, and the “place” or positioning within the food outlet.^11^, ^12^ Other research that has explored elements likely to improve the healthiness of food environments has expanded on the 4Ps framework by identifying additional intervention targets in food retail settings. These additional intervention targets, supplementing the 4Ps to make up the 7Ps, include the “people” (or employees) who sell products, the “processes” by which products are delivered to the consumer (including, for example, the standard side dishes or condiments served with a meal), and “partnerships” between retailers and other stakeholders.^13^ Engaging food and beverage retailers to intervene across the 7Ps and change the consumer nutrition environment at food retail outlets remains a challenge.^14^, ^15^, ^16^ In particular, previous studies have identified that retailers frequently express concerns that healthy food and beverage interventions will compromise business commercial viability and that appropriate healthier product alternatives are not readily available to stock.^17^, ^18^ Additional challenges previously identified by retailers include lack of perceived consumer demand for healthy food, confusion in what constitutes a “healthy” food offering, and fear of profit loss.^16^

Food outlet‐level accreditation schemes (hereafter referred to as “schemes”) represent one strategy for engaging with retailers to improve the healthiness of food and beverage outlets.^10^ Using predefined criteria^10^ to assess organizational practice(s), such schemes may increase the healthiness of consumer purchases within food outlet settings by changing, among other food environment characteristics, the relative availability, placement, promotion, and price of healthier options.^19^, ^20^, ^21^, ^22^ However, we are only aware of one systematic review that has included an analysis of the impact of such schemes. The previous review, conducted in 2017, focused on the impact of accreditation schemes on practices to promote healthier ready‐to‐eat meals, finding increases in healthier catering practices and availability of healthier options.^10^ Both included studies were of a weak study design. No systematic reviews have comprehensively examined the impact of schemes on a full range of outcomes of interest to retailers and policymakers, including changes to outlet practices; impacts on consumer purchasing behavior; retailer and customer awareness; understanding, satisfaction, and support of schemes; and barriers and enablers to successful scheme uptake and maintenance. A greater understanding of the implementation and impact of schemes has the potential to lead to improved scheme design that could result in greater uptake of these initiatives by retailers and policymakers and increase the effectiveness of these schemes to improve population health and nutrition outcomes.

The aim of this systematic review was to assess the impact of nutrition‐related food retail outlet‐level accreditation schemes on food retail outlet practices and customer purchasing behavior. We also aimed to identify the reported barriers and enablers to scheme implementation, including scheme uptake (the proportion of retailers signing up for a scheme) and certification (the proportion of retailers meeting scheme requirements).

## 2 METHODS

### 2.1 Search strategy

The selection, analysis, and reporting of the results for this study were conducted in accordance with the Preferred Reporting Items for Systematic Reviews and Meta‐Analyses (PRISMA) guidelines^23^ (see Table S1 for the completed checklist). An initial scoping review was conducted to identify key studies, which were used to inform the final search strategy. The search protocol was registered online with PROSPERO on April 3, 2021 (CRD42021240769).

Electronic databases (Embase, EBSCO Medline, EBSCO Global Health, EBSCO Business Source Complete, and ERIC) and grey literature (Google Advanced Search, first 100 results) were systematically searched to identify studies that related to retail outlet‐level healthy food and beverage accreditation schemes. The four hedge terms (“food outlets,” “accreditation schemes,” “nutrition,” and “outcomes of interest”) were combined with the operator “AND,” and within each hedge, specific search terms were combined with the operator “OR.” The included EBSCO Medline‐specific search terms are shown in Table 1. Boolean search operators were adjusted for each database searched (see Table S2 for full search strategies for each database). Backward searching of reference lists of included articles and forward searching of articles that have cited included articles were undertaken to optimize the search process. A research librarian was consulted to develop this search strategy.

**TABLE 1:** Search terms for EBSCO Medline.

Hedge 1: Food outlets	Hedge 2: Accreditation schemes	Hedge 3: Nutrition	Hedge 4: Outcomes of interest
Outlet OR	Award OR	Food* OR	Perception* OR
Retail* OR	Accreditation OR	Drink* OR	Consum* OR
Store OR	Program OR	Beverage* OR	Purchas* OR
Restaurant OR	Initiative OR	Heath* OR	Sale* OR
Café OR	Recognition OR	Nutrition	Uptake OR
Cafeteria OR	Scheme		Adopt* OR
Canteen OR			Practice* OR
Cater* OR			Availability OR
Takeaway			Compliance OR
			Implement OR
			Sustainability

### 2.2 Inclusion criteria

To be included, studies must have reported on the adoption of, or compliance with, nutrition‐related food outlet‐level schemes, or the impact of participation on outcomes of interest (Table 2). Accreditation schemes were defined as interventions or programs, including awards, accreditation, or other recognition, based on an assessment of organizational practice(s) using predefined criteria.^10^ To be included, the outlet, organization, or the scheme had to be related to food provision or food retail, and the scheme had to include an element related to food or nutrition, including nutrition schemes, alcohol provision policies, or food sustainability policies. We included observational and experimental study designs.

**TABLE 2:** Review inclusion and exclusion criteria.

Study parameters	Include	Exclude
Population	Food retail outlets, including cafés, bars, restaurants, convenience stores, supermarkets, schools, hospitals, workplaces, and other facilities where food is sold or provided	Non‐food retail outlets
Intervention	Schemes included elements related to food and/or nutrition: interventions or programs, including awards, accreditation, or other recognition, based on an assessment of organizational practice(s) using predefined criteria	Schemes that did not include elements related to food, beverages, or nutrition. Schemes that included no components that were likely to improve the nutritional value or healthfulness of food purchasing were excluded
Comparator	N/A	N/A
Outcomes	Any outcome resulting from adoption of, or compliance to, nutrition‐related food outlet‐level accreditations, including scheme uptake or changes to the food environment, effect on customer purchasing or dietary intake, customer perspectives, retailer perspectives, commercial viability, cost, cost‐effectiveness, and process outcomes	N/A
Study design	All observational and experimental research	Ambiguous research designs, theoretical studies, and methods papers
Publication type	Original research papers	Opinion pieces, reviews, protocols, and abstracts
Language	English	Languages other than English
Date of publication	All dates	N/A

### 2.3 Study selection process

Following the database searches, article titles, keywords, and abstracts were imported into COVIDENCE for removal of duplicates and subsequent screening by two independent authors (OH and TBR). Articles deemed to be potentially relevant based on title and abstract content had their full texts screened against the inclusion/exclusion criteria by two independent authors (OH and JA). Any disagreements were resolved through discussions with a third author (MB). For the grey literature search, the first 10 pages of search results (100 results) were screened by two authors (OH and MB) to identify potentially relevant records.

### 2.4 Data extraction

Two authors independently extracted data from included studies using a standard template in Microsoft Excel (for each included study, any two of OH, SS, JA, MB, and TBR completed data extraction). This information was cross‐checked, and any disagreements were resolved through discussion with a third author. Extracted data included bibliographic data, study design, study funding, scheme criteria required to receive accreditation, value proposition to retailers, governance (engagement and recruitment, enforcement, assessment, and monitoring processes), implementation (uptake, certification, provision of support and resources, responsibility for implementation, and monitoring), outcomes (effect on customer purchasing or eating patterns, business outcomes [customer perspectives, retailer perspectives, and commercial viability],^24^ cost‐effectiveness, and process outcomes), program costs/resources, scheme duration, and sustainability. Scheme uptake was defined as the proportion of retailers who committed to participate in the scheme relative to the number of retailers that were invited to participate. Scheme certification was defined as the proportion of retailers who received the scheme award or accreditation relative to the total number of retailers who committed to participate. The aspects of the food environment that were targeted by schemes were defined according to the 7Ps: “product,” “promotion,” “price,” “place,” “processes,” “people,” and “partnerships.”^11^, ^12^, ^13^, ^25^

### 2.5 Quality appraisal

The quality of included quantitative, qualitative, and mixed methods studies was determined using the Mixed Methods Appraisal Tool (MMAT) (Table S3).^26^ The MMAT is a critical appraisal tool designed to appraise the methodological quality of five study categories: qualitative research, randomized controlled trials, nonrandomized studies, quantitative descriptive studies, and mixed methods studies. Five criteria exist for qualitative research, randomized controlled trials, nonrandomized studies, and quantitative descriptive studies, allowing these study types to be scored out of 5. Mixed methods studies are scored according to the qualitative research criteria, the relevant quantitative research criteria, and an additional mixed methods criterion, allowing these studies to be scored out of 15.

Although the original MMAT does not provide cut‐offs for high‐, medium‐, and low‐quality studies, we applied previously applied cut‐offs^26^ to identify studies that scored ≥80% as high‐quality studies, studies that scored 50%–80% as medium‐quality studies, and studies that score ≤50% as low‐quality studies.

### 2.6 Data synthesis

Results were narratively synthesized because of anticipated heterogeneity of populations and outcomes.^27^ Schemes were grouped by the target food outlet type: (i) restaurants, cafés, and bars; (ii) convenience and corner stores; (iii) schools and childcare centers; (iv) hospitals and healthcare settings; (v) other general workplaces (henceforth referred to as “workplaces”), or (vi) multiple different settings targeted with the same broad accreditation criteria.

Scheme findings were summarized according to (i) scheme characteristics, including food environment targets, scheme governance, support offered for scheme implementation, and scheme monitoring and compliance; (ii) scheme outcomes, including uptake, certification, impact on purchasing, customer perspectives, and retailer perspectives; and (iii) barriers and enablers to scheme implementation. Data synthesis included the vote‐counting method following Cochrane advice^28^ to summarize effect estimates for scheme outcomes of interest as in (ii) above. Schemes were counted as having an overall “positive” or “negative”/“neutral” impact. Schemes were considered as having a “positive” impact if the study supported the award scheme: increased uptake of healthy retail practices, increased the healthiness of customer purchases, was supported by customers or retailers, or was associated with commercial outcomes favorable to retailers. Outcomes were also classified as “positive” if over 50% of the counted variable were deemed as having been an improvement to food environments, or customer or retailer behaviors or perspectives.

Barriers and enablers to scheme implementation were extracted if they were explicitly identified by the original authors as barriers and enablers in the results sections of included studies. The authors of the current review then inductively coded each barrier and enabler according to similar constructs (e.g., lack of retailer time for implementation). These barriers and enablers were then grouped into themes.

## 3 RESULTS

**FIGURE 1:**
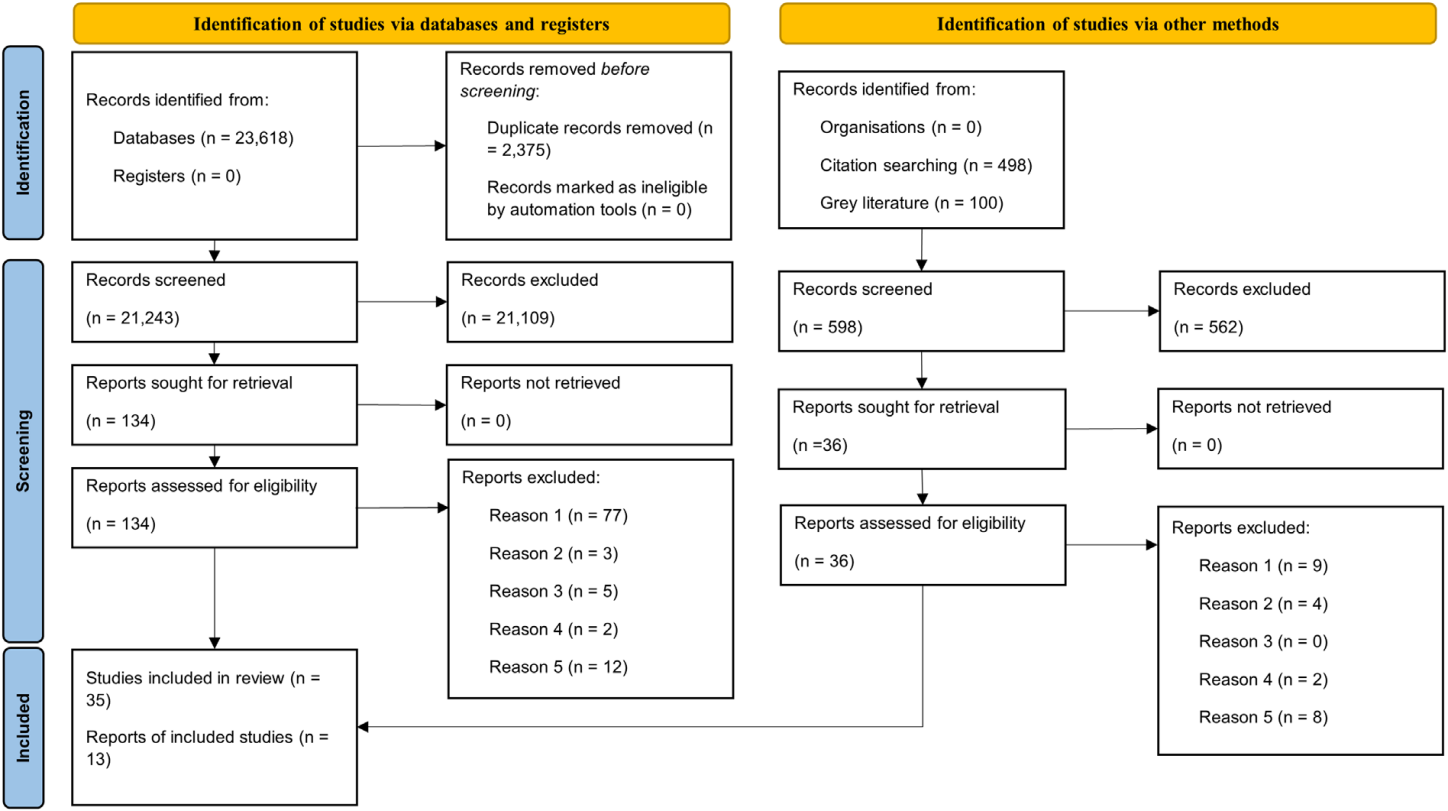
Search strategy PRISMA flow diagram. Reason 1: Article excluded because it did not meet the definition of an accreditation scheme. Reason 2: Article excluded because the accreditation scheme did not include food outlet‐level implementation. Reason 3: Article excluded because the accreditation scheme did not include a healthy food and/or beverage component. Reason 4: Article excluded because it did not include an outcome of interest. Reason 5: Article excluded for reasons outside of the above.

Figure 1 shows the PRISMA flow diagram for this systematic review.^23^ The systematic literature search identified 21,943 records, of which 2052 were excluded as duplicates, leaving 19,891 records to be screened. 19,772 records were excluded based on irrelevant title and abstract. 119 records were read in full and assessed against the exclusion criteria, with 35 records found to be eligible for data extraction and narrative synthesis. An additional 13 records were identified through grey literature searching, and forward and backward searching of included articles, resulting in a total of 48 records being included in the synthesis.

### 3.1 Study characteristics

The date of publication for included studies ranged from 2004 to 2021 (Table S4). Of the 48 included studies, one was a randomized controlled trial, one was a nonrandomized quantitative experimental study, 22 were descriptive observational quantitative studies, seven were observational qualitative studies, and 18 were observational mixed methods studies. As determined by the MMAT,^26^ 18 of the included studies were high quality, 15 were medium quality, and 15 were of low quality. Details of the MMAT scoring for each study are found in Table S5.

### 3.2 Accreditation scheme characteristics

The 48 included studies covered 26 different schemes (Table S6). Of these 26 schemes, 14 were based in the USA, five each were based in Canada and the UK, and two were based in Australia. Nine schemes targeted restaurants, nine targeted convenience and corner stores, three targeted schools and childcare settings, one targeted hospitals, one targeted workplaces, and four targeted multiple retailer types.

### 3.3 Accreditation scheme governance

Of the 26 identified schemes, 22 had their governance mechanism, or their overarching managing body, described.^19^, ^21^, ^22^, ^29^, ^30^, ^31^, ^32^, ^33^, ^34^, ^35^, ^36^, ^37^, ^38^, ^39^, ^40^, ^41^, ^42^, ^43^, ^44^, ^45^, ^46^, ^47^, ^48^, ^49^, ^50^, ^51^, ^52^, ^53^, ^54^, ^55^, ^56^, ^57^, ^58^, ^59^, ^60^, ^61^, ^62^, ^63^ Ten schemes were managed by a coalition or collective of stakeholders. Coalition members commonly included community groups, nongovernment organizations (NGOs), retail representatives, government agencies, and academics. For example, the ¡Por Vida! Initiative was governed by the San Antonio's Healthy Restaurant Coalition, which includes members from the San Antonio Metropolitan Health District, the San Antonio Restaurant Association, and the San Antonio Dietetic Association.^29^, ^30^, ^31^

Ten schemes were managed by either a local or national government agency. For example, the Healthy Bodegas initiative was coordinated by the New York City Department of Health and Mental Hygiene and was funded by the New York City Center for Economic Opportunity.^40^ One scheme, the Heart Smart Restaurant Program, was led by an NGO (the Heart and Stroke Foundation of Canada).^34^ Finally, one scheme, Waupaca Eating Smart, was led by an academic team.^52^, ^53^

### 3.4 Accreditation scheme monitoring

Of the 26 identified schemes, 17 had a specific monitoring and compliance strategy reported.^19^, ^21^, ^29^, ^30^, ^31^, ^32^, ^33^, ^34^, ^35^, ^36^, ^37^, ^38^, ^39^, ^40^, ^41^, ^42^, ^43^, ^44^, ^45^, ^46^, ^47^, ^48^, ^49^, ^50^, ^52^, ^53^, ^57^, ^58^, ^59^, ^60^, ^61^, ^63^ Monitoring of compliance was most commonly the responsibility of either governance team staff members (such as environmental health officers for the Healthier Catering Commitment^19^, ^64^ or trained volunteers and local community groups (such as the food justice leaders employed under Healthy Retail San Francisco.^37^, ^38^

As all schemes were voluntary, no penalties for noncompliance were reported. In some cases, monitored outlets were provided with additional support, such as additional funding offered for further implementation of the Healthy HotSpot initiative.^42^, ^43^

### 3.5 Accreditation scheme food classification criteria

Of the 26 included schemes, 20 described the rationale for the criteria by which foods and beverages were classified as healthy or not healthy. Eight schemes derived their criteria from existing nutrition guidelines, such as population‐wide dietary guidelines or school nutrition guidelines.^20^, ^29^, ^30^, ^45^, ^46^, ^47^, ^48^, ^49^, ^51^, ^52^, ^53^, ^54^, ^55^, ^56^, ^62^ The criteria for two schemes were developed following a review of the literature on existing accreditation schemes in similar settings to form the basis for new criteria.^21^, ^36^, ^39^ Three schemes classified foods as healthy/unhealthy based on specific nutrient values (commonly sodium, sugar, and fat), although in all three cases, the nutrient cut‐offs were not provided.^35^, ^41^, ^57^, ^58^, ^59^, ^65^, ^66^, ^67^, ^68^ Three schemes targeted specific categories of healthy foods (fruits and vegetables) without targeting “unhealthy” foods and beverages.^37^, ^38^, ^44^, ^63^ Two schemes adapted the criteria used for previous initiatives or schemes for use as a framework for their own criteria.^19^, ^33^, ^34^, ^64^ Two schemes did not rely on existing criteria and instead consulted with stakeholders, including governing bodies, retailers, and customers, in the development of their criteria.^32^, ^40^ Of the 19 schemes, only the ¡Por Vida! Initiative provided nutrient cut‐offs (derived from the *2005 Dietary Guidelines for Americans*) for the energy, total fat, saturated fat, trans fat, and sodium contents of targeted meals.^29^, ^30^ Accreditation scheme food classification criteria were applied in various ways but were most commonly used to encourage product or menu reformulation to meet a set standard and designate which products should be relatively more or less available, and which products should be promoted.

### 3.6 Accreditation scheme environmental changes

All components of the food environment (as characterized by the 7Ps^11^, ^12^, ^13^, ^25^) were targeted by at least one identified scheme. All identified schemes targeted some aspects of “product.” Two common examples of this included changing the available food products to make menus healthier and offering smaller portion sizes. For example, the US ¡Por Vida! Initiative encouraged restaurants to alter menu items to meet a range of nutrient criteria,^21^ whereas US “Shape Up Somerville: Eat Smart, Play Hard” included requirements for provision of smaller portion sizes in restaurants.^20^

Seventeen schemes encouraged food retail outlets to implement “promotions.” This was commonly the use of posters, table tents, and other promotional materials to promote healthy eating and/or the scheme itself. Eleven schemes attempted to change the “people” aspect of food environments, most commonly by training outlet staff in the preparation and upselling of healthier foods and beverages. Ten schemes encouraged food retail outlets to change their “processes.” This took the form of fundamental shifts in the way that food was offered at outlets, without changing the food that was actually available. For example, under the “Healthier Catering Commitment”, UK fast food outlets were encouraged to no longer add salt to menu items (instead allowing customers to do so themselves).^19^

Seven schemes targeted the “place” aspect of food environments, often by changing store layouts to make healthier items more accessible or prominent. For example, “Choose Health LA Restaurants” required that drinking water be easily accessible in restaurants.^21^ Six schemes leveraged “partnerships” to promote healthier customer choices. This included partnering outlets with local councils to provide support with promoting healthy eating and partnering outlets with other health‐promoting businesses. For example, corner stores that signed up for the “Healthy HotSpot” initiative received support in the form of community outreach and assistance with engagement with local institutions.^42^ Finally, one scheme encouraged food retail outlets to change their “prices” to make healthier foods and beverages relatively more affordable.

### 3.7 Accreditation scheme implementation support

Of the 26 identified schemes, 24 were reported as offering outlets support to implement the scheme.^19^, ^21^, ^22^, ^29^, ^30^, ^31^, ^32^, ^33^, ^34^, ^35^, ^36^, ^37^, ^38^, ^39^, ^40^, ^41^, ^42^, ^43^, ^44^, ^45^, ^46^, ^47^, ^48^, ^49^, ^50^, ^51^, ^52^, ^53^, ^54^, ^55^, ^56^, ^57^, ^58^, ^59^, ^60^, ^62^, ^63^, ^66^, ^69^ Support took multiple forms, including provision of promotional materials, retail staff training, and provision of financial support or equipment. Just two schemes offered promotional support alone^20^, ^63^; all other schemes also offered technical assistance or additional resources.

Technical assistance was frequently offered to retailers. This commonly took the form of retail staff training, assistance with any menu changes, and/or nutritional classification of available products by a trained dietitian. All restaurants participating in the Choose Health LA Restaurants program were offered technical assistance with the process for applying to participate in the program and achieve certification, and any menu changes.^21^, ^36^

Where schemes included promotion of healthy eating, provision of promotional materials was common. Restaurants participating in the Shape Up Somerville: Eat Smart, Play Hard intervention were supplied with 1‐in. stickers that could be placed on existing menus, boards, or signs and were given assistance in designing menu inserts, a 4‐in. window decal, and laminated signs and table tents listing the “Shape Up Approved” criteria.^20^

Three schemes offered more substantive financial support to either act as an incentive or to assist with implementation. This included direct financial contributions to retailers and also provision of more expensive equipment, such as food storage and display items. For example, the English Department of Health provided 50% of the costs for a new chill cabinet for fresh fruits and vegetables to retail outlets participating in the Change4Life convenience store intervention.^44^

### 3.8 Accreditation scheme outcomes

All 26 schemes had elements of their impact on food environments, customer purchases, and/or customer and/or retailer perceptions reported (Table S7).

### 3.9 Accreditation scheme uptake

Accreditation scheme uptake refers to the number or proportion of retailers that elected to participate in a scheme but does not reflect the number that achieved the scheme requirements to be certified. Fourteen schemes had data available on scheme uptake, of which, seven reported the number of retail outlets eligible to participate (allowing uptake to be calculated). Average uptake across these seven schemes was 65%. Three studies of high‐quality reported scheme uptake of range 47% to 88%. At the lower end, approximately 43% of restaurants in Somerville signed up for Shape‐up Somerville.^20^ Highest scheme uptake was seen in South Australia, where 44/50 (88%) of daycare centers expressed interest in signing up for Start Right–Eat Right, although the recruitment and engagement strategy for this scheme was not reported on.^46^

### 3.10 Accreditation scheme certification

Accreditation scheme certification refers to the proportion of participating schemes that achieved the scheme requirements to be certified.

**FIGURE 2:**
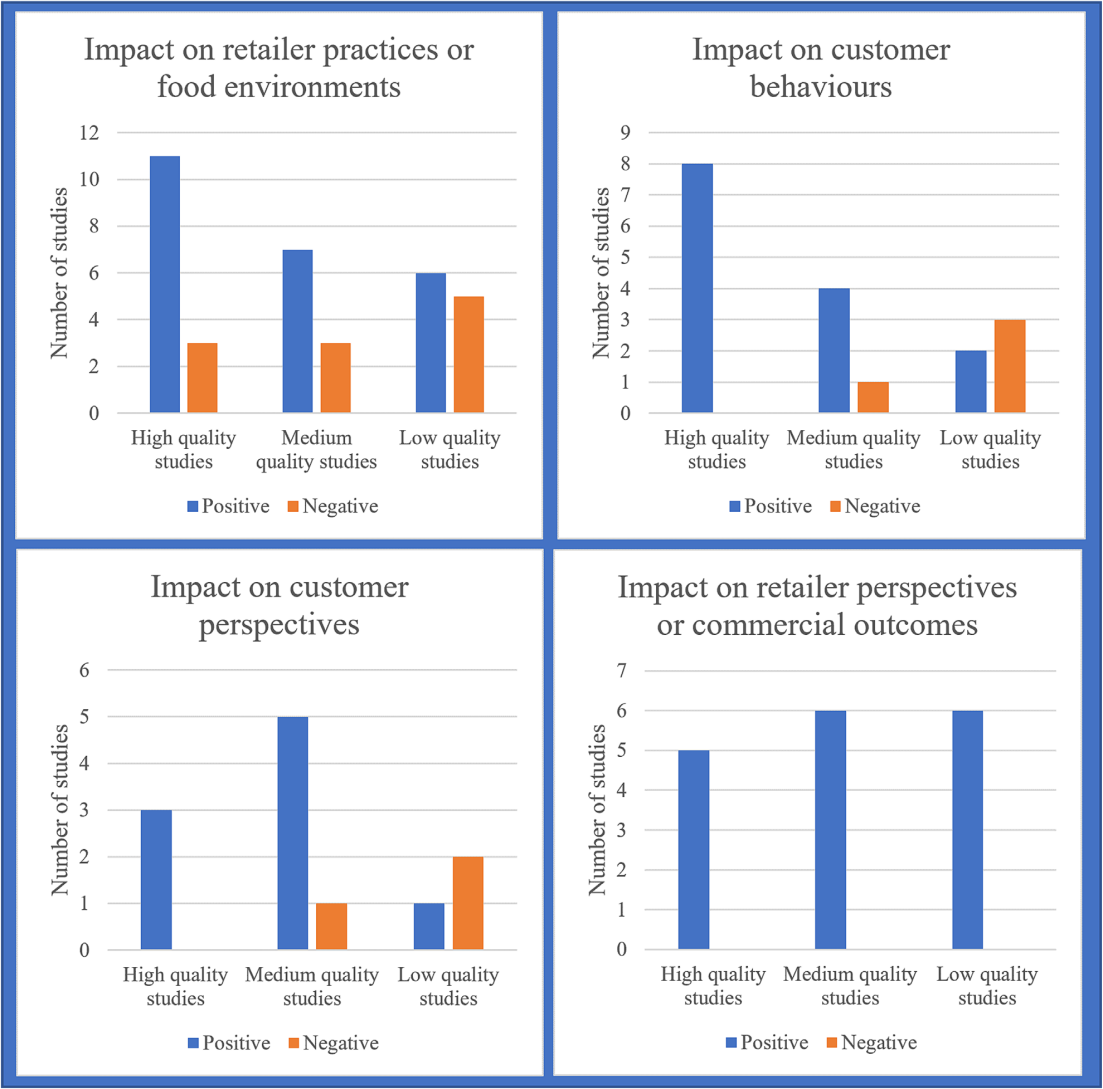
Accreditation scheme impact counting for included studies.

Thirty‐five included studies reported on scheme impact on the healthiness of food outlets (Figure 2 and Table S8), of which 14 were classified as high‐quality studies. These 35 studies included all 26 included schemes. Of these 35 studies, 24 reported an overall positive change to the food retail environment. Of the 11 studies that did not report an overall positive change, all reported on accreditation schemes implemented in restaurants or multiple settings. This pattern was the same among the 14 high‐quality studies that reported on scheme impact on the healthiness of food outlets. All schemes reported some improvements to the healthiness of food environments at some food outlets, even when certification rates were low or unreported. This included all high‐quality studies.^20^, ^41^, ^42^, ^44^, ^45^, ^48^, ^49^, ^50^, ^54^, ^56^, ^64^, ^69^, ^70^, ^71^

Fourteen schemes reported on the proportion of retailers who achieved scheme certification (nine schemes were reported on in high‐quality studies). The mean certification rate among these schemes was 64% (range 6%–100%). The mean proportion of certified retailers reported in high‐quality studies was 54% (range 6%–100%).^20^, ^44^, ^45^, ^49^, ^50^, ^54^, ^56^, ^67^, ^69^ Schemes that provided multiple levels of certification (such as bronze, silver, and gold certification levels) commonly had a higher proportion of certified retailers as businesses could aim for a lower level and still be considered “certified.” For example, 24 private hospitals joined the Healthy Hospital Food Initiative. Nine (38%) reached “gold” accreditation (achieving nutrition standards in all four domains), seven (29%) reached “silver” accreditation (achieving nutrition standards in two or three domains), three (13%) reached “bronze” accreditation (achieving nutrition standards in one domain), and five (21%) did not implement any standards.^50^ Therefore, the Healthy Hospital Food Initiative had an overall proportion of certified retailers of 79%, although just 38% of hospitals reached full (“gold”) certification.

Another observed driver of the proportion of certified retailers was the number of retail outlets recruited for participation. Schemes with fewer participating outlets tended to provide greater levels of support and had a higher proportion of certified retailers. Waupaca Eating Smart recruited seven restaurants and two supermarkets to participate, and all outlets implemented some Waupaca Eating Smart activities.^52^ In comparison, 2989 schools were recruited to participate in the Food For Life Partnership, and 192 achieved any certification.^56^

### 3.11 Accreditation scheme impact on eating patterns and purchasing

Eighteen included studies reported on scheme impact on customer eating patterns and purchasing (Figure 2 and Table S8), of which eight were classified as high‐quality studies. Of these 18 studies, 14 reported an overall positive change to customer eating patterns. Of the four studies that did not report an overall positive change, all reported on accreditation schemes implemented in restaurants or multiple settings. All eight high‐quality studies reported a positive change to customer eating patterns or purchases. These 18 studies reported on the impact on the healthiness of customer purchasing for 14 schemes.

Of these 14 schemes, 10 increased customer purchases of targeted healthier items,^20^, ^37^, ^39^, ^41^, ^44^, ^48^, ^52^, ^56^, ^60^, ^69^ two had no impact on customer purchasing,^19^, ^33^, ^34^ and two provided some limited evidence of scheme impact on the healthiness of customer purchasing.^40^, ^57^, ^58^, ^59^, ^65^, ^66^, ^67^ Although it was rarely reported, three schemes reported reduced purchasing of unhealthy items (Healthy2Go, Heartbeat Award scheme, and Start Right–Eat Right).^39^, ^48^, ^65^ In the case of the Start Right–Eat Right scheme, this was reported in a high‐quality study.^48^ Scheme impact on customer purchasing varied by outlet type. Just two of the six restaurant schemes that provided data available on customer purchases reported increases to the healthiness of customer purchases.^19^, ^34^ In comparison, 83% of the eight schemes that targeted convenience stores reported increases to the healthiness of customer purchases.^37^, ^39^, ^41^, ^44^, ^69^ Most schemes that targeted multiple settings showed some evidence of improvements to the healthiness of consumer purchases,^52^, ^56^, ^60^ although the widely promoted Heartbeat Award scheme exhibited minimal evidence of impact.^57^, ^58^, ^59^, ^65^, ^66^, ^67^ A positive impact on customer purchases was reported for the Heartbeat Award scheme in a high‐quality study.^59^ The impact of scheme participation on customer nutrient intakes was reported by a high‐quality study for just one scheme: Start Right–Eat Right.^48^ This scheme was associated with improvements in both food and nutrient intakes among children attending participating centers.

### 3.12 Accreditation scheme retailer perspectives and commercial outcomes

Seventeen included studies reported on scheme impact on retailer perspectives or commercial outcomes (Figure 2 and Table S8), of which five were classified as high‐quality studies. All of these studies reported an overall positive change to retailer perspectives. These 17 studies reported retailers' perspectives for 17 schemes. Across the schemes, indicators of retailer satisfaction with schemes were high. This included a high proportion of retailers planning to continue with their scheme (4/4 schemes),^22^, ^33^, ^39^, ^52^, ^53^ a high proportion of participating retailers recommending participation to nonparticipating retailers (1/1 scheme),^32^ and high retailer awareness of, support for, and understanding of schemes (5/5 schemes).^20^, ^33^, ^44^, ^47^, ^52^, ^53^ A common reason provided for scheme participation was to provide local communities with healthy and nutritious foods.^37^, ^38^, ^43^, ^49^, ^54^, ^55^ Among restaurant owners who did not participate in an advertised scheme, the most common reasons were lack of understanding about how to participate, lack of time, and concerns about loss of revenue.^19^, ^72^ Concerns relating to financial viability were reported in the high‐quality studies by Lynch et al.,^63^ McDaniel et al.,^38^ and Boelsen‐Robinson et al.^64^

### 3.13 Accreditation scheme customer awareness and perspectives

Twelve included studies reported on scheme impact on customer awareness and perspectives (Figure 2 and Table S8), of which three were classified as high‐quality studies. Of these 12 studies, nine reported an overall positive change to either customer awareness or perspectives (but not always both). Of the three studies that did not report an overall positive change, one reported on an accreditation scheme implemented in restaurants, and two reported on an accreditation scheme implemented in multiple settings. The three high‐quality studies reported a positive change to customer awareness or perspectives. The 12 studies reported on customers' perspectives for 11 schemes. Customer support for and satisfaction with schemes, where measured, was consistently high (4/4 schemes).^29^, ^30^, ^35^, ^39^, ^51^ Conversely, customer awareness and understanding of schemes was usually low (4/5 schemes), and this was associated with low scheme impact on the healthiness of customer purchases.^34^, ^36^, ^41^, ^57^, ^58^ Conversely, Project FIT saw increases in customer understanding of the scheme and associated increases in purchasing of healthier foods.^69^

### 3.14 Barriers and enablers to accreditation scheme implementation

Barriers and enablers to implementation were reported for 20 schemes (Table 3). In general, elements that were associated with scheme uptake, certification, and impact on customer purchases explicitly considered the perspectives, value to, and support of the retailer.

**TABLE 3:** Summary of barriers and enablers of accreditation scheme implementation.

Scheme characteristics	Enablers	Barriers
Scheme criteria	Flexible delivery allowing retailers to select which scheme criteria they wished to meet^19^, ^20^, ^32^, ^64^	Eligibility criteria to join exclude some businesses^19^, ^64^
	Tiered scheme with multiple levels of criteria^19^, ^64^	Short initiative timeframe^42^
	Feasible, culturally acceptable, and tailored delivery to businesses^19^, ^64^	Scheme not worthwhile given retailer food options^70^
	Some businesses only have small changes to make to meet criteria^19^, ^64^	Resource and time intensity of delivery^19^, ^32^, ^58^, ^63^, ^64^, ^68^
	Incorporating pilot scheme learnings to ensure compatibility with business practices^47^	Requirements for staff training and support^47^, ^53^
	Low resource and space requirements^53^	
Retailer recruitment	Convenience of applying to participate^70^, ^72^	Misunderstandings about how to qualify for the program^72^
	Existing scheme easy to pick up^19^, ^64^, ^70^	Slow approval process^42^
	Public recognition of certification^19^, ^58^, ^64^	Lack of retailer engagement^20^, ^37^, ^38^, ^44^
	Scheme provides a competitive advantage^54^	Fear of loss of revenue^20^, ^62^, ^63^, ^72^
	Highly motivated retailers^32^, ^37^, ^38^, ^39^, ^43^, ^58^, ^62^	
	Available facilities^54^	
Governance	Well‐publicized program ensuring that retailers were aware it was available^20^, ^33^, ^37^, ^38^, ^62^	Program not well publicized^33^
	Strong program communication and feedback to retailers that had already enlisted, from the governing body^50^	Slow equipment ordering and delivering^42^
	Retailer engagement and ownership of program^53^, ^71^	Requirements for franchise approval to participate^37^, ^38^, ^58^, ^62^, ^72^
	Existing relationships between retailers and governing bodies^19^, ^64^	Sometimes weak existing relationships between retailers and scheme governing body^19^, ^64^
	Collaborative efforts and partnerships which draw on shared expertise^19^, ^29^, ^30^, ^43^, ^47^, ^64^	Poor communication and contact with food business owners^19^, ^44^, ^64^
Implementation support	Alternative payment for food handler training^72^	Cost of implementation^29^, ^30^, ^44^, ^46^, ^47^, ^49^, ^58^, ^68^, ^70^, ^71^
	Strategic targeting to make efficient use of time^19^, ^64^	Lack of time^20^, ^37^, ^38^, ^46^, ^47^, ^72^
	Provision and sharing of resources^19^, ^37^, ^38^, ^60^, ^64^	Lack of resources^37^, ^38^, ^40^
	Funding for delivery^19^, ^37^, ^38^, ^62^, ^64^	Low technical skills and competing responsibilities of retailer^32^, ^62^
	Dietitian and environmental health officer support^19^, ^32^, ^50^, ^64^	Difficulties in sourcing healthy products^29^, ^30^, ^32^, ^37^, ^38^, ^39^, ^47^, ^50^, ^56^, ^63^
	Franchise executives' approval to participate^37^, ^38^, ^58^, ^72^	Fresh food wastage^40^, ^44^
	Government buy‐in to scheme success^47^, ^50^	Low organization awareness^49^, ^70^
		Personnel changes^37^, ^38^, ^42^, ^46^, ^62^
		Unsuitability of provided promotional materials^20^, ^33^, ^40^
		Low availability or quality of promotional materials^44^
Monitoring and evaluation	Flexibility in the assessment of restaurants, with multiple assessment options (such as phone and face‐to‐face interviews)^72^	Lack of evaluation data^37^, ^38^, ^50^
	Visibility of positive outcomes and results^47^	
Customer support for and awareness of scheme	The opportunity for positive advertising^72^	Owners fear loss of customers^19^, ^64^
	Customer demand for healthy foods and beverages^72^	Lack of customer demand^37^, ^38^, ^53^, ^71^
	Increased customer interest in health^19^, ^64^	
	Customer engagement with healthy food retail accreditation scheme^58^	
Other	Shared culture and strong community relationships^40^, ^43^	Challenges exacerbated for businesses in areas of deprivation^19^, ^44^, ^64^

Several key and overarching enablers of scheme implementation were identified. Pilot schemes, supported by research, were noted as useful for ensuring compatibility with businesses and facilitating implementation.^47^ Such compatibility included ensuring that scheme criteria aligned with what was feasible for retailers, developing a convenient scheme recruitment strategy, ensuring strong communication and engagement from governing bodies with retailers, and providing ample support with implementation. Retailers were more likely to participate in a scheme that they perceived as easy to pick up^72^ and where success was publicly recognized and led to a competitive advantage.^19^, ^54^, ^58^, ^64^, ^73^ Scheme criteria and support could also be tailored to enable implementation by including alternative payment for food handler training;^72^ strategic targeting to make efficient use of time;^19^, ^64^ provision and sharing of resources between retailers, community organizations, and governing bodies;^19^, ^37^, ^38^, ^60^, ^64^ provision of funding for implementation;^19^, ^37^, ^38^, ^64^ support provided by dietitians and environmental health officers;^19^, ^32^, ^50^, ^64^ and a low scheme requirement for resources and physical space.^53^

Barriers to scheme implementation were also identified. These included promotional activities or materials being unsuitable for display in some businesses,^20^, ^33^, ^40^ poor publicizing of a scheme,^33^ a slow process for the ordering and delivering of scheme equipment and materials,^42^ and low levels of retailer time and availability,^20^, ^37^, ^38^, ^46^, ^47^, ^72^ resources,^37^, ^38^, ^40^ or technical skills^32^ to implement an accreditation scheme. Further, some schemes were relatively time or resource intensive to implement.^19^, ^32^, ^58^, ^64^, ^68^ The requirement to provide healthy products was also seen as a barrier, as some retailers reported difficulties in sourcing these,^29^, ^30^, ^32^, ^37^, ^38^, ^39^, ^47^, ^50^, ^56^ and others reported fresh food wastage.^40^, ^44^ Where retailers believed there was low customer demand for healthier options,^37^, ^38^, ^53^, ^71^ feared potential loss of revenue^20^, ^72^ or business,^19^, ^64^ or had concerns about the cost of implementation,^29^, ^30^, ^44^, ^46^, ^47^, ^49^, ^58^, ^68^, ^71^ scheme uptake and certification were frequently impacted.

## 4 DISCUSSION

This systematic review of 26 schemes reported in 46 studies found that nutrition‐related food outlet‐level accreditation schemes were associated with improvements in outlet practices and customer purchasing behavior. Of the 26 schemes, nine targeted restaurants, nine targeted convenience and corner stores, three targeted schools and childcare settings, one targeted hospitals, one targeted workplaces, and four targeted multiple retailer types. All included schemes targeted improvements in the healthiness of products available, as well as other elements of the food environment. The healthiness of customer purchases improved across many setting types (convenience stores, schools, and hospitals), but evidence from restaurant schemes was mixed. Schemes were most commonly governed by either a coalition or collective of stakeholders (10 schemes) or a local or national government agency (11 schemes), although some schemes were managed by an NGO (one scheme) or academic team (one scheme).

Key factors associated with scheme uptake and implementation identified included support provided for scheme implementation and maintenance, flexibility for retailers in meeting scheme criteria, and motivation of retailers and staff. Average uptake across the seven schemes reporting uptake was 65% (range 43%–88%). Customer purchases of targeted healthier items increased in 10 of the 14 schemes, which reported on scheme impact on the healthiness of customer purchasing. Only the Start Right–Eat Right early childhood education scheme was evaluated for impact on nutritional intake (rather than using purchasing as a proxy measure for healthier consumption).^48^ With heterogeneity in outcomes, it was difficult to assess the overall magnitude of scheme impact on purchasing behavior.

A meta‐analysis^74^ of healthy food and beverage interventions in real‐life grocery stores found that promotion‐based interventions (*k* = 15) were associated with an effect size of 0.10 (95%CI 0.02, 0.18) on target food purchases, similar to “prompting” interventions like menu labeling (*k* = 12) 0.14 (95%CI 0.09, 0.19). This is a smaller effect size than was found in three included high‐quality studies reported on various indicators relating to the purchase and consumption of products targeted by accreditation schemes. Paluta et al.^41^ reported that the number of healthy items sold per month at participating corner stores increased from 133.3 to 309.5 items following the implementation of Fresh Foods Here. Likewise, 38%–46.3% of customers shopping at corner stores participating in FIT stores self‐reported that they had increased their purchasing of grains, proteins, low‐fat dairy, or fruits and vegetables compared with the previous year.

Multicomponent retail food environment interventions, and interventions that make larger changes to the food environment (targeting multiple of the 7Ps, rather than just “products”), have been more consistently associated with favorable impacts on customer purchasing behaviour.^10^, ^11^ Schemes that target multiple aspects of the retail environment may therefore be more effective at improving the healthiness of customer purchases. Tiered schemes, with multiple levels to achieve, were associated with increased scheme uptake,^64^ healthy changes to the food environment,^19^, ^39^, ^40^ and increases in customer purchases of healthier items.^40^ There was also evidence that retailers might “stall” at lower scheme levels and fail to make further health‐promoting changes.^40^ The evidence synthesized here suggests that such schemes should incorporate greater support and incentives for retailers to make further changes to achieve higher levels of certification. In the case of the Healthy HotSpot Initiative, retailers were awarded additional funding in response to further changes to the store environment.^42^, ^43^

Across the included schemes, customer support for schemes was high, although awareness and understanding were generally low. Low scheme awareness was associated with a low impact on customer purchases. Behavior economic theory suggests that consumer awareness of an intervention is not required for changes in consumer behavior.^75^ Indicators of retailer satisfaction with schemes were also high, and qualitative research echoed previous research findings that retailers' ability to contribute to customer and community wellbeing was an important motivator.^24^ It is likely that at least some of the favorable retailer feedback is related to a participation bias, as retailers more supportive of healthy food environments are more likely to adopt such schemes. As evaluations did not typically compare participating and nonparticipating retailers, it is not known which scheme formats have the strongest recruitment potential, nor how to expand the reach of schemes to a greater diversity of retailers (and communities).

The higher proportion of convenience store schemes reporting increases to the healthiness of customer purchases (83%) compared with restaurant schemes (33%) may be related to the small number of included restaurant schemes, or perhaps variation in the accreditation criteria used in different settings. As convenience stores typically sell packaged nonperishable food, these outlets may be able to offer direct substitutes for less healthy alternatives more easily than restaurants, which may require more substantive changes in food storage, cooking, and ordering practices. Several schemes targeting restaurants noted the complexity and time intensity of full nutritional analyses of menus by registered dietitians.^29^, ^30^, ^50^ Schemes using simple food‐based criteria (e.g., restrictions on deep‐fried foods and encouraging fresh fruit and vegetables) may therefore be more easily understood by retailers and less costly to monitor but may also only be appropriate for certain retailer types, such as those offering substantive quantities of fresh fruits and vegetables. We are aware of only one previous systematic review that examined the findings of two award schemes^10^ but was unable to draw general conclusions about this type of retail intervention or factors that are likely to be associated with scheme impacts. More work is needed to further understand the characteristics of impactful schemes in these settings and whether scheme characteristics including accreditation criteria or implementation support may need to differ by setting.

In the current review, the most common enablers reported to increase uptake and implementation of schemes included a tiered approach to scheme participation with multiple levels of achievement, public recognition of certification, and provision and sharing of resources including support provided by people skilled in nutrition science to implement changes, franchise or retailer executives' approval to participate, high retailer motivation, and collaborative efforts and partnerships drawing on shared expertise. The most common barriers reported included difficulties in sourcing healthy products; lack of retailer or franchise engagement; and cost, resourcing, and time intensity of implementation. These enablers and barriers were largely similar to those identified in a recent review of reviews of factors influencing implementation of healthy food retail interventions,^16^ which found key influences including “Retailer knowledge, skills and preferences regarding healthy food (and interventions),” “Organisational Support (Control and Ownership over Food Store Supplies),” “Resources (Staff, Time, Capital),” and “Establishing Partnerships” with a range of stakeholders. Further reviews of factors affecting implementation of healthy food retail interventions have also emphasized difficulties in maintaining a constant supply of healthy alternatives at an affordable price.^76^, ^77^ The current review did not make direct comparisons between the characteristics or the barriers and enablers of accreditation schemes compared with other healthy food retail initiatives. It is likely that the “offer” to the retailer, including public recognition or certification, is particularly important in the context of accreditation schemes.

### 4.1 Implications for practice

This systematic review provides the first synthesis of evidence that food retail accreditation schemes may be effective in improving the healthiness of some consumer food environments. Although none of the studies included in this review examined changes in energy intake or weight outcomes, accreditation schemes are unlikely to have a significant impact on population weight when used alone. Addressing the obesogenic food environment is widely acknowledged to require changes throughout the food system.^78^ Our findings suggest that accreditation schemes may be an effective mechanism of engaging commercial retailers in healthy food retail change, an otherwise hard‐to‐reach group.^16^

**FIGURE 3:**
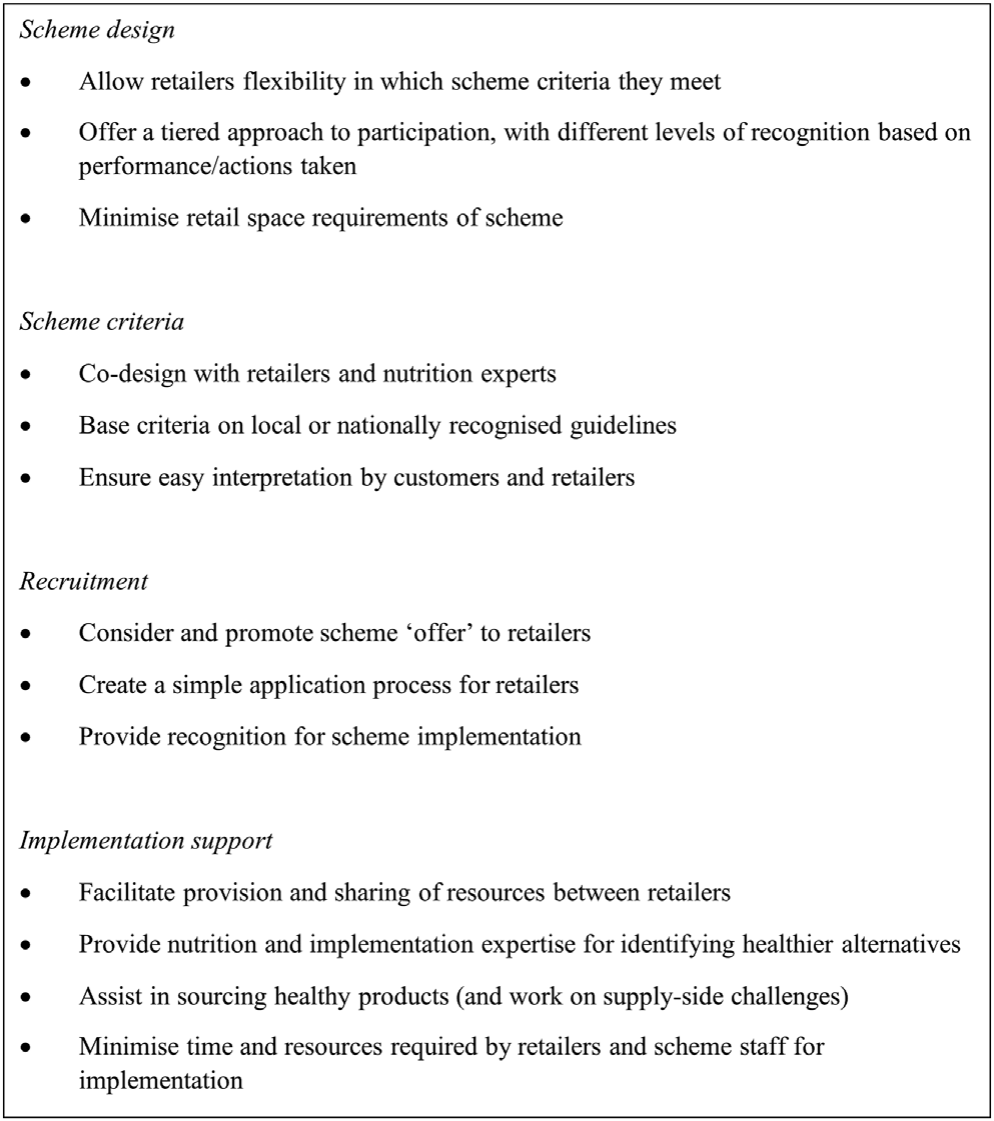
Recommendations for practitioners to support the implementation of healthy food retail accreditation schemes.

This review provides a number of key lessons for those designing and supporting healthy food retail accreditation schemes. We have formulated recommendations for governing bodies to guide scheme development and implementation based on the barriers and enablers reported and key common elements of scheme criteria, design, implementation support, monitoring and evaluation, and governance (see Figure 3).

### 4.2 Strengths and limitations of studies included in the review

As determined by the MMAT (26), 17 included studies were high quality, 13 included studies were medium quality, and 16 included studies were of low quality. Of the 16 low‐quality studies, 10 were quantitative descriptive studies. These commonly scored low because of a lack of reporting on methodological characteristics. This highlights the importance of ensuring that methodological approaches to quantitative evaluations of accreditation schemes are robustly described. Additionally, 10 of the 17 high‐quality studies were quantitative descriptive studies.

Our review was restricted to articles in English but did not exclude studies based on scheme location. Nineteen of the 26 schemes identified were located in North America, and all schemes were located in English‐speaking OECD‐member countries. This may limit the generalizability of the results to high‐income countries, which may have different retail environments and consumer expectations.^79^ Further, 16 of the 26 identified schemes targeted restaurants or convenience stores. The results presented herein may be more generalizable to these settings. Despite this, schemes were shown to be effective at improving food environments and customer choices in other settings.

A small number of included studies used validated tools to evaluate outcomes including changes in the healthiness of the food environment (e.g., NEMS‐CS^80^). Most included studies used unvalidated and unstandardized tools to measure outcomes. Researchers should be encouraged to make use of existing validated tools to measure implementation (e.g., the Implementation Outcome Repository^81^). Only the Start Right–Eat Right early childhood education scheme was evaluated for impact on nutrition intake,^48^ instead changes in customer consumption were inferred from changes in sales or, most commonly, customer or retailer‐reported changes in purchasing behavior. Future research should measure the impact of schemes on customer eating patterns. This review did not identify any cost‐effectiveness evidence for food retail accreditation schemes. Future research is required to determine the value for money of these interventions from various perspectives.

### 4.3 Methodological strengths and limitations of the review

This study is the first review of food retail accreditation schemes that captured outcomes needed in the design and execution of schemes by policy maker and retailers. The inclusion of both grey and peer‐reviewed literature and studies with a range of qualitative, quantitative, and mixed methods designs and a range of outcomes facilitated discussion of the holistic impacts and considerations for implementing such schemes. Heterogeneity in study design and scheme design increased the difficulty in synthesizing associations between scheme characteristics and outcomes. As the review did not include retail interventions other than accreditation schemes, we were unable to make direct comparisons with the effectiveness of different approaches to incentivizing and supporting retailers to make food environment changes. Finally, we did not explicitly focus on the process of scheme development, which may limit the application of these results to future scheme development.

## 5 CONCLUSIONS

Nutrition‐related food outlet‐level accreditation schemes represent a promising mechanism for engaging food retailers to improve the healthiness of food retail environments. Accreditation schemes may offer different incentives and accountability mechanisms, although it is unclear if they are more effective than other kinds of healthy food retail interventions. Schemes appear to be influenced by many of the same barriers and enablers as other healthy food retail initiatives, emphasizing the need to address structural barriers to retailer changes including the supply of healthier food products. Further research is required on the impacts of accreditation scheme participation on the healthiness of customer purchases and population eating patterns.

## CONFLICT OF INTEREST STATEMENT

None.

## Supporting information


**Table S1:** Preferred Reporting Items for Systematic Reviews and Meta‐Analyses (PRISMA) guidelines
**Table S2:** Full search strategies for each database
**Table S3:** Mixed Methods Appraisal Tool (MMAT)
**Table S4:** Included study characteristics
**Table S5:** Mixed Methods Appraisal Tool (MMAT) results
**Table S6:** Accreditation scheme characteristics
**Table S7:** Outcomes of included accreditation schemes
**Table S8:** Accreditation scheme impact counting for included studies
