# Supplementary material for: The implementation and effectiveness of outlet‐level healthy food and beverage accreditation schemes: A systematic review
Source: Obes Rev. 2023 Feb 8;24(4):e13556. doi: 10.1111/obr.13556 (PMC10909553; doi:10.1111/obr.13556)
Supplement: Supplementary file 1 — Table S1: Preferred Reporting Items for Systematic Reviews and Meta‐Analyses (PRISMA) guidelines Table S2: Full search strategies for each database Table S3: Mixed Methods Appraisal Tool (MMAT) Table S4: Included study characteristics Table S5: Mixed Methods Appraisal Tool (MMAT) results Table S6: Accreditation scheme characteristics Table S7: Outcomes of included accreditation schemes Table S8: Accreditation scheme impact counting for included studies [file OBR-24-e13556-s001.pdf]

The implementation and effectiveness of outlet-level healthy food and beverage accreditation schemes: a systematic scoping review

Supplementary files

Oliver Huse[1], Sally Schultz[1], Tara Boelsen-Robinson[1], Jaithri Ananthapavan[1,2], Anna Peeters[1], Gary Sacks[1], Miranda R Blake[1]

[1] Deakin University, Geelong, Australia. Global Centre for Preventive Health and Nutrition (GLOBE), Institute for Health Transformation, Faculty of Health.
[2] Deakin University, Geelong, Australia. Deakin Health Economics, Institute for Health Transformation, Faculty of Health.

Corresponding Author:
Dr Miranda Blake
miranda.blake@deakin.edu.au
Deakin University
221 Burwood Hwy
Burwood VIC 3125
Australia

Appendix 1: Preferred Reporting Items for Systematic Reviews and Meta-Analyses (PRISMA) guidelines

Section and Topic	Item #	Checklist item	Location where item is reported
**TITLE**			
Title	1	Identify the report as a systematic review.	Page 1
**ABSTRACT**			
Abstract	2	See the PRISMA 2020 for Abstracts checklist.	Page 1
**INTRODUCTION**			
Rationale	3	Describe the rationale for the review in the context of existing knowledge.	Page 1-2
Objectives	4	Provide an explicit statement of the objective(s) or question(s) the review addresses.	Page 2
**METHODS**			
Eligibility criteria	5	Specify the inclusion and exclusion criteria for the review and how studies were grouped for the syntheses.	Page 2
Information sources	6	Specify all databases, registers, websites, organisations, reference lists and other sources searched or consulted to identify studies. Specify the date when each source was last searched or consulted.	Page 2
Search strategy	7	Present the full search strategies for all databases, registers and websites, including any filters and limits used.	Appendix 2
Selection process	8	Specify the methods used to decide whether a study met the inclusion criteria of the review, including how many reviewers screened each record and each report retrieved, whether they worked independently, and if applicable, details of automation tools used in the process.	Page 2-3
Data collection process	9	Specify the methods used to collect data from reports, including how many reviewers collected data from each report, whether they worked independently, any processes for obtaining or confirming data from study investigators, and if applicable, details of automation tools used in the process.	Page 3
Data items	10a	List and define all outcomes for which data were sought. Specify whether all results that were compatible with each outcome domain in each study were sought (e.g. for all measures, time points, analyses), and if not, the methods used to decide which results to collect.	Page 3
	10b	List and define all other variables for which data were sought (e.g. participant and intervention characteristics, funding sources). Describe any assumptions made about any missing or unclear information.	Page 3
Study risk of bias assessment	11	Specify the methods used to assess risk of bias in the included studies, including details of the tool(s) used, how many reviewers assessed each study and whether they worked independently, and if applicable, details of automation tools used in the process.	Page 4
Effect measures	12	Specify for each outcome the effect measure(s) (e.g. risk ratio, mean difference) used in the synthesis or presentation of results.	N/A
Synthesis methods	13a	Describe the processes used to decide which studies were eligible for each synthesis (e.g. tabulating the study intervention characteristics and comparing against the planned groups for each synthesis (item #5)).	Page 3
	13b	Describe any methods required to prepare the data for presentation or synthesis, such as handling of missing summary statistics, or data conversions.	N/A
	13c	Describe any methods used to tabulate or visually display results of individual studies and syntheses.	N/A
	13d	Describe any methods used to synthesize results and provide a rationale for the choice(s). If meta-analysis was performed, describe the model(s), method(s) to identify the presence and extent of statistical heterogeneity, and software package(s) used.	Page 4
	13e	Describe any methods used to explore possible causes of heterogeneity among study results (e.g. subgroup analysis, meta-regression).	N/A
	13f	Describe any sensitivity analyses conducted to assess robustness of the synthesized results.	N/A
Reporting bias assessment	14	Describe any methods used to assess risk of bias due to missing results in a synthesis (arising from reporting biases).	N/A

Section and Topic	Item #	Checklist item	Location where item is reported
Certainty assessment	15	Describe any methods used to assess certainty (or confidence) in the body of evidence for an outcome.	N/A
**RESULTS**			
Study selection	16a	Describe the results of the search and selection process, from the number of records identified in the search to the number of studies included in the review, ideally using a flow diagram.	Page 4
	16b	Cite studies that might appear to meet the inclusion criteria, but which were excluded, and explain why they were excluded.	N/A
Study characteristics	17	Cite each included study and present its characteristics.	Appendix 4
Risk of bias in studies	18	Present assessments of risk of bias for each included study.	Appendix 5
Results of individual studies	19	For all outcomes, present, for each study: (a) summary statistics for each group (where appropriate) and (b) an effect estimate and its precision (e.g. confidence/credible interval), ideally using structured tables or plots.	Pages 5-8; Appendices 6-7
Results of syntheses	20a	For each synthesis, briefly summarise the characteristics and risk of bias among contributing studies.	Page 5-8
	20b	Present results of all statistical syntheses conducted. If meta-analysis was done, present for each the summary estimate and its precision (e.g. confidence/credible interval) and measures of statistical heterogeneity. If comparing groups, describe the direction of the effect.	N/A
	20c	Present results of all investigations of possible causes of heterogeneity among study results.	Pages 5-8
	20d	Present results of all sensitivity analyses conducted to assess the robustness of the synthesized results.	N/A
Reporting biases	21	Present assessments of risk of bias due to missing results (arising from reporting biases) for each synthesis assessed.	N/A
Certainty of evidence	22	Present assessments of certainty (or confidence) in the body of evidence for each outcome assessed.	N/A
**DISCUSSION**			
Discussion	23a	Provide a general interpretation of the results in the context of other evidence.	Pages 10-12
	23b	Discuss any limitations of the evidence included in the review.	Page 11
	23c	Discuss any limitations of the review processes used.	Page 12
	23d	Discuss implications of the results for practice, policy, and future research.	Page 11; Figure 3
**OTHER INFORMATION**			
Registration and protocol	24a	Provide registration information for the review, including register name and registration number, or state that the review was not registered.	Page 2
	24b	Indicate where the review protocol can be accessed, or state that a protocol was not prepared.	Page 2
	24c	Describe and explain any amendments to information provided at registration or in the protocol.	N/A
Support	25	Describe sources of financial or non-financial support for the review, and the role of the funders or sponsors in the review.	Page 1
Competing interests	26	Declare any competing interests of review authors.	page 2
Availability of data, code and other materials	27	Report which of the following are publicly available and where they can be found: template data collection forms; data extracted from included studies; data used for all analyses; analytic code; any other materials used in the review.	N/A

Appendix 2: Full search strategies for each database

Hedge	EBSCO Business Source Complete	EBSCO Global Health	EBSCO Medline	Embase	ERIC
**Food outlets**	Outlet OR<br>Retail* OR<br>Store OR<br>Restaurant OR<br>Café OR<br>Cafeteria OR<br>Canteen OR<br>Cater* OR<br>Takeaway	Outlet OR<br>Retail* OR<br>Store OR<br>Restaurant OR<br>Café OR<br>Cafeteria OR<br>Canteen OR<br>Cater* OR<br>Takeaway	Outlet OR<br>Retail* OR<br>Store OR<br>Restaurant OR<br>Café OR<br>Cafeteria OR<br>Canteen OR<br>Cater* OR<br>Takeaway	Outlet OR<br>Retail* OR<br>Store OR<br>Restaurant OR<br>Café OR<br>Cafeteria OR<br>Canteen OR<br>Cater* OR<br>Takeaway	Outlet OR<br>Retail* OR<br>Store OR<br>Restaurant OR<br>Café OR<br>Cafeteria OR<br>Canteen OR<br>Cater* OR<br>Takeaway
**Accreditation schemes**	Award OR<br>Accreditation OR<br>Program OR<br>Initiative OR<br>Recognition OR<br>Scheme	Award OR<br>Accreditation OR<br>Program OR<br>Initiative OR<br>Recognition OR<br>Scheme	Award OR<br>Accreditation OR<br>Program OR<br>Initiative OR<br>Recognition OR<br>Scheme	Award OR<br>Accreditation OR<br>Program OR<br>Initiative OR<br>Recognition OR<br>Scheme	Award OR<br>Accreditation OR<br>Program OR<br>Initiative OR<br>Recognition OR<br>Scheme
**Nutrition**	Food* OR<br>Drink* OR<br>Beverage* OR<br>Heath* OR<br>Nutrition	Food* OR<br>Drink* OR<br>Beverage* OR<br>Heath* OR<br>Nutrition	Food* OR<br>Drink* OR<br>Beverage* OR<br>Heath* OR<br>Nutrition	Food* OR<br>Drink* OR<br>Beverage* OR<br>Heath* OR<br>Nutrition	Food* OR<br>Drink* OR<br>Beverage* OR<br>Heath* OR<br>Nutrition
**Outcomes of interest**	Perception* OR<br>Consum* OR<br>Purchas* OR<br>Sale* OR<br>Uptake OR<br>Adopt* OR<br>Practice* OR<br>Availability OR<br>Compliance OR<br>Implement OR<br>Sustainability	Perception* OR<br>Consum* OR<br>Purchas* OR<br>Sale* OR<br>Uptake OR<br>Adopt* OR<br>Practice* OR<br>Availability OR<br>Compliance OR<br>Implement OR<br>Sustainability	Perception* OR<br>Consum* OR<br>Purchas* OR<br>Sale* OR<br>Uptake OR<br>Adopt* OR<br>Practice* OR<br>Availability OR<br>Compliance OR<br>Implement OR<br>Sustainability	Perception* OR<br>Consum* OR<br>Purchas* OR<br>Sale* OR<br>Uptake OR<br>Adopt* OR<br>Practice* OR<br>Availability OR<br>Compliance OR<br>Implement OR<br>Sustainability	Perception* OR<br>Consum* OR<br>Purchas* OR<br>Sale* OR<br>Uptake OR<br>Adopt* OR<br>Practice* OR<br>Availability OR<br>Compliance OR<br>Implement OR<br>Sustainability

Appendix 3: Mixed Methods Appraisal Tool (MMAT)

Category of study designs	Methodological quality criteria	Responses			
		Yes	No	Can't tell	Comments
Screening questions (for all types)	S1. Are there clear research questions?				
	S2. Do the collected data allow to address the research questions?				
	Further appraisal may not be feasible or appropriate when the answer is 'No' or 'Can't tell' to one or both screening questions.				
1. Qualitative	1.1. Is the qualitative approach appropriate to answer the research question?				
	1.2. Are the qualitative data collection methods adequate to address the research question?				
	1.3. Are the findings adequately derived from the data?				
	1.4. Is the interpretation of results sufficiently substantiated by data?				
	1.5. Is there coherence between qualitative data sources, collection, analysis and interpretation?				
2. Quantitative randomized controlled trials	2.1. Is randomization appropriately performed?				
	2.2. Are the groups comparable at baseline?				
	2.3. Are there complete outcome data?				
	2.4. Are outcome assessors blinded to the intervention provided?				
	2.5 Did the participants adhere to the assigned intervention?				
3. Quantitative non-randomized	3.1. Are the participants representative of the target population?				
	3.2. Are measurements appropriate regarding both the outcome and intervention (or exposure)?				
	3.3. Are there complete outcome data?				
	3.4. Are the confounders accounted for in the design and analysis?				
	3.5. During the study period, is the intervention administered (or exposure occurred) as intended?				
4. Quantitative descriptive	4.1. Is the sampling strategy relevant to address the research question?				
	4.2. Is the sample representative of the target population?				
	4.3. Are the measurements appropriate?				
	4.4. Is the risk of nonresponse bias low?				
	4.5. Is the statistical analysis appropriate to answer the research question?				
5. Mixed methods	5.1. Is there an adequate rationale for using a mixed methods design to address the research question?				
	5.2. Are the different components of the study effectively integrated to answer the research question?				
	5.3. Are the outputs of the integration of qualitative and quantitative components adequately interpreted?				
	5.4. Are divergences and inconsistencies between quantitative and qualitative results adequately addressed?				
	5.5. Do the different components of the study adhere to the quality criteria of each tradition of the methods involved?				

Appendix 4: Included study characteristics

First author surname; Year of publication; Intervention "name"	Study design; Study timing; Length of pre-intervention; intervention; follow-up	Retail outlet sample size (Response rate if relevant and reported); recruitment method if reported	Individual respondent sample size (Response rate if relevant and reported); recruitment method if reported	Data source/s used- Measurement instrument/ tool	Analytical methods	Funding	Study quality
Adams; 2012; Change4Life Convenience Store intervention (1)	Mixed methods; post only; cross sectional	Opt-out consent for store visits; 15/17 demonstration stores (88%), 59/70 roll-out stores (84%)	10 retailers participated in interviews; Purposive sampling	Data collection proforma to measure fidelity of marketing tools and fresh fruits and vegetable (FFV) availability.<br>Key informant interviews with retailers	*Food environment:* Descriptive statistics.<br>*Interviews:* Thematic analysis	National Institute for Health Research	High
Andreyeva; 2018; USDA Child and Adult Care Food Program (CACFP) (2)	Quantitative descriptive; post only; cross-sectional	87 childcare centres; Purposive sampling of CACFP participating centres (48%)	Not relevant	Adapted version of the validated Rudd Center Child Care Director's survey assessed childcare centre practices.	Chi-square and t-tests compared proportion of centres meeting CACFP guidelines, teacher feeding behaviours and preschool nutrition practices in CACFP participating vs non-CACFP participating (n=256, response rate 56%) centres.	USDA National Institute of Food and Agriculture<br><br>Robert Wood Johnson Foundation	High
Andreyeva; 2022; USDA Child and Adult Care Food Program (CACFP) (3)	Quantitative descriptive; post only; cross-sectional	231 childcare centres; Purposive sampling of 96 CACFP participating centres (55%).	Not relevant	Retailer survey assessed childcare centre practices and CACFP characteristics and outcomes.<br>Foregone federal meal reimbursements were estimated from administrative data.	T-tests compared proportion of centres meeting CACFP guidelines, teacher feeding behaviours and preschool nutrition practices in CACFP participating vs non-CACFP participating (n=135, response rate 35%) centres.<br>Logistic regressions assessed predictors of CACFP participation, including centre and	Child Health and Development Institute of Connecticut<br><br>Children's Fund of Connecticut<br><br>Connecticut Health Foundation	High

First author surname; Year of publication; Intervention "name"	Study design; Study timing; Length of pre-intervention; intervention; follow-up	Retail outlet sample size (Response rate if relevant and reported); recruitment method if reported	Individual respondent sample size (Response rate if relevant and reported); recruitment method if reported	Data source/s used- Measurement instrument/ tool	Analytical methods	Funding	Study quality
					neighbourhood characteristics.	Newman's Own Foundation	
Bagwell; 2013; Healthier Catering Commitment (HCC) (4)	Mixed methods; cross-sectional<br><br>Online surveys: post only<br><br>Interviews: post only<br><br>Focus groups: pre-post	Online survey: 77 businesses across 12 London boroughs; Convenience sampling of restaurants participating in HCC	In-depth interviews: 10 managers and 28 customers from 5 businesses<br>Focus groups: 2 environmental health officers, 1 nutritionist, 1 public health practitioner	Online surveys with retailers; telephone and face-to-face interviews with retailers and customers; focus groups of implementation support staff	Not reported	Greater London Authority<br><br>Chartered Institute of Environmental Health<br><br>Inner North West London Primary Care Trusts	Medium
Bell; 2012; Start Right - Eat Right (SRER) (5)	Quantitative descriptive; post only; cross-sectional	110 day-care centres; Simple random sampling	Not relevant	16-item survey assessed:<br>- Mealtime environment<br>- Communicating with families about nutrition<br>- Respondent characteristics	*Survey:* Awarded centres (n=36) were compared to trained but not awarded (n=42) and untrained (n=32) centres.<br>T-tests assessed difference in outcomes by SRER participation.	SA Health	High
Bell; 2015; Start Right - Eat Right (SRER) (6)	Quantitative descriptive; post only; cross-sectional	20 Early Learning long day care centres; Convenience sample of centres scheduled to participate in SRER over a set time period	Not relevant	Dietitian observed and recorded children's dietary intake at morning tea, lunch and afternoon tea.<br>Nutrition policy, menu and food environment data were collected at baseline and follow-up.	*Dietitian observations:* Mann–Whitney U test and T-tests compared dietary data post-centre participation with population guidelines.<br>*Food environment and menu:* Descriptive statistics presented for centre compliance to scheme criteria.	University of South Australia<br><br>SA Health	High

**First author surname; Year of publication; Intervention "name"**	**Study design; Study timing; Length of pre-intervention; intervention; follow-up**	**Retail outlet sample size (Response rate if relevant and reported); recruitment method if reported**	**Individual respondent sample size (Response rate if relevant and reported); recruitment method if reported**	**Data source/s used- Measurement instrument/ tool**	**Analytical methods**	**Funding**	**Study quality**
Biediger-Friedman; 2014; ¡Por Vida! menu labelling initiative (7)	Quantitative descriptive; post only; cross-sectional	Pilot: 1 restaurant.<br>Full initiative: 7 restaurant brands with >75 outlets; Voluntary responses	95 restaurant patrons; Recruitment method not reported	Customer surveys (pilot), media coverage and attendance at events (full intervention launch)	Not reported	Texas Department of State Health Services	Low
Boelsen-Robinson; 2020; Healthier Catering Commitment (HCC) (8)	Qualitative; post only; cross-sectional	Not relevant	22 individuals overseeing the HCC; Purposive sampling	Semi-structured interviews	*Interviews:* Thematic analysis	No study-specific funding reported	High
Brown; 2017; Savvy Diner (9)	Mixed methods; post only; cross-sectional	22 independent restaurants; Purposive sampling	9 retailers participated in exit interviews; Convenience sample	Retailer survey assessed willingness to participate in a menu labelling pilot program.<br>Exit interviews with restaurant operators.<br>Focus groups and interviews conducted with scheme staff.	*Surveys:* Not reported (assessed willingness to participate prior to recruitment)<br>*Interviews and focus groups:* Thematic analysis	Toronto Public Health	Low
Dannefer; 2012; Healthy Bodegas Initiative (10)	Quantitative descriptive; pre-post; 7 months	60 stores; Purposive sampling of participating stores	Convenience sample of 228 customers exiting a subset of 10 stores were recruited to participate in a survey post-implementation	Observation of store alignment with criteria.<br>Retailer surveys<br>Customer surveys	*Survey responses and store alignment with criteria:* T-tests compared pre- to post-implementation	New York City Center for Economic Opportunity<br><br>New York City Department of Health and Mental Hygiene	Medium
Dawson; 2006; Eat Smart! Workplace Cafeteria Program (11)	Mixed-methods; post-only; cross-sectional; 4 months after implementation	1 hospital	258 (all) staff members not on leave (51%); Convenience sample	Questionnaires sent to hospital staff. Questions covered:<br>(i) Cafeteria use<br>(ii) Program awareness<br>(iii) Program attitudes	*Surveys:* Descriptive statistics.<br>Thematic analysis of open-ended questions	Public Health Research Education and Development program, Public Health and Community	Medium

First author surname; Year of publication; Intervention "name"	Study design; Study timing; Length of pre-intervention; intervention; follow-up	Retail outlet sample size (Response rate if relevant and reported); recruitment method if reported	Individual respondent sample size (Response rate if relevant and reported); recruitment method if reported	Data source/s used- Measurement instrument/ tool	Analytical methods	Funding	Study quality
				(iv) Self-reported change in eating behaviour<br>5. Suggestions to improve program		Services, Hamilton, Ontario	
DeWeese; 2016; United States Department of Agriculture's Special Supplemental Nutrition Program for Women, Infants, and Children (WIC). Supplemental Nutrition Assistance Program (SNAP) (12)	Quantitative descriptive; post only; cross-sectional	Total 325 stores including purposive sampling of 43 ‘upgraded’ stores. Random sampling of 282 ‘non-upgraded’ stores	Not relevant	Stores' healthy food offerings were assessed using the Nutrition Environment Measures Survey for Corner Stores (NEMS-CS) and the newly developed short-form corner store audit tool (SCAT)	Multiple regression compared NEMS-CS and SCAT scores in WIC stores vs SNAP stores. Adjusted for sales volume, store size, and area-level education, income and ethnicity.	National Institute of Food and Agriculture<br><br>National Institute of Child Health and Human Development	Medium
Dombrowski; 2019; Healthy HotSpot (13)	Mixed methods; post only; cross-sectional	21 stores; Purposive sampling of all stores who participated in Healthy HotSpot initiative	Convenience sample of customers in 6 of 8 participating communities recruited a focus group.	Short customer and staff survey informed interviews with retailers and focus groups with customers	*Interviews and focus groups:* Thematic analysis	Public Health Institute of Metropolitan Chicago<br><br>Cook County Department of Public Health	Medium
Dwivedi; 1999; Heart Smart Restaurant Program (14)	Quantitative descriptive; post only; cross-sectional	142 restaurants; Convenience sampling	Not relevant	Retailer surveys	Not reported	Ontario Heart Health Action Program, Heart Health Resource Centre<br><br>Regional Municipality of Ottawa-Carlton	Low
Dwyer; 2004; Eat Smart! Ontario's	Qualitative; post only; cross-sectional	35 restaurants; Purposive sampling	35 restaurant operators who did not participate in the program	Structured interview guide covering:<br>(i) Program benefits	*Interviews:* Thematic analysis	Canadian Foundation for	Medium

First author surname; Year of publication; Intervention "name"	Study design; Study timing; Length of pre-intervention; intervention; follow-up	Retail outlet sample size (Response rate if relevant and reported); recruitment method if reported	Individual respondent sample size (Response rate if relevant and reported); recruitment method if reported	Data source/s used- Measurement instrument/ tool	Analytical methods	Funding	Study quality
Healthy Restaurant Program (15)		with assistance of scheme governing body		(ii) Barriers to participation<br>(iii) Desired support<br>(iv) Increasing program participation		Dietetic Research	
Economos; 2009; Shape Up Somerville: Eat Smart, Play Hard (16)	Mixed methods; post only; cross-sectional	74 restaurants; Purposive sampling to targeting family friendly restaurants with the potential for healthy changes	Not relevant	Scheme development informed by short interviews with retailers.<br>Compliance assessed through site visits.<br>Retailer surveys assessed compliance, customer feedback, customer purchasing, and benefits of participation.	Not reported	Centers for Disease Control and Prevention, Atlanta, Georgia	High
Escaron; 2016; Waupaca Eating Smart (17)	Quantitative descriptive; data collected at 4-6 months into intervention period and 2 months after intervention period.	7 restaurants, 2 grocery stores; All participating retailers participated in study	Not relevant	Checklist to record scheme implementation<br><br>Face-to-face surveys with retailers at the end of the intervention.	No comparison; Descriptive statistics for all measures	University of Wisconsin School of Medicine and Public Health	Low
Fitzpatrick; 1997; Fresh Choice (18)	Mixed methods; post only; cross-sectional	8 stores; Purposive sampling of participating stores	686 store customers surveyed; Convenience sample<br><br>8 customers interviewed (1 per store); Purposive sampling	Customer survey questionnaire asking customer to identify fresh choices.<br>Semi-structured interviews with customers	*Surveys:* T-tests comparing satisfaction between low-fat and regular menu items; adjust for order (item type, meal/non-meal), gender, age, frequency of and reason for eating out<br>*Interviews:* Thematic analysis	Vancouver Health Department<br><br>Restaurant and Foodservices Association of Greater Vancouver	High
Gase; 2015; Choose Health LA Restaurants (19)	Quantitative descriptive; pre-post;	17 restaurant chains, 42 restaurant sites; All restaurant sites with	Not relevant	Standardized data collection form assessed portions, prices and	*Menu changes:* Descriptive statistics	Centers for Disease Control and	Medium

First author surname; Year of publication; Intervention "name"	Study design; Study timing; Length of pre-intervention; intervention; follow-up	Retail outlet sample size (Response rate if relevant and reported); recruitment method if reported	Individual respondent sample size (Response rate if relevant and reported); recruitment method if reported	Data source/s used- Measurement instrument/ tool	Analytical methods	Funding	Study quality
	cross-sectional over 1 year	applications approved between a set timeframe were assessed		labelling, and children's meals.		Prevention<br><br>First 5 LA	
Gase; 2016; Choose Health LA Restaurants (20)	Mixed methods; post only; cross sectional	Not reported	802 adult customers; Online recruitment	Online survey consisted of 75 questions in 4 sections: demographics, health behaviours and knowledge, perceptions of and behaviours at restaurants, awareness of and perceptions of the Choose Health LA	*Media articles:* Content analysis<br><br>*Online survey:* Descriptive statistics; survey weights applied	Centers for Disease Control and Prevention	Medium
Gray; 2015; Food for Life Partnership (21)	Qualitative; post only; cross-sectional	7 hospitals	Not reported	Semi-structured interviews with retailers	*Interviews:* Thematic analysis	Not reported	Low
Gray; 2017; Food for Life Partnership (22)	Qualitative; post-only; cross-sectional	3 participating national health service trusts	25 hospital staff; Purposive sampling	Semi-structured interviews and analysis of program documentation (meeting minutes, strategic plans and reports)	*Interviews and documents:* Thematic analysis	Not reported	High
Green; 1993; Heart Smart Restaurant Program (23)	Quantitative descriptive; post only; cross-sectional	499 participants in Saskatoon, 500 participants in Regina; Random sampling of residents in 2 cities	Not reported	Telephone survey assessed consumer awareness, understanding and utilization of the program, and restaurant compliance.	Not reported	Heart and Stroke Foundation of Saskatchewan	Low
Holdsworth; 1997; Heartbeat Award (HBA) (24)	Quantitative descriptive; post only; cross-sectional	11 establishments; Convenience sample of establishments in Leicestershire participating in HBA	271 customers; Convenience sample	Customer survey assessed: customer awareness, understanding, satisfaction with HBA scheme	*Surveys:* Chi-squared test assessed differences in participants by age, gender, and social class	Leicestershire Health	Medium
Holdsworth; 1999; Heartbeat Award (HBA) (25)	Quantitative descriptive; post only; cross-sectional	23 establishments (12 workplaces, 11 public eating establishments); Purposive sample of all	Not relevant	Background questionnaire assessed outlet menu practices (number of items, menu	No analysis (raw data only)	Leicestershire Health	High

First author surname; Year of publication; Intervention "name"	Study design; Study timing; Length of pre-intervention; intervention; follow-up	Retail outlet sample size (Response rate if relevant and reported); recruitment method if reported	Individual respondent sample size (Response rate if relevant and reported); recruitment method if reported	Data source/s used- Measurement instrument/ tool	Analytical methods	Funding	Study quality
		Leicestershire HBA establishments		cycle, healthy practices).<br><br>HBA checklist assessed the type of foods that the canteen had available.			
Holdsworth; 2000; Heartbeat Award (HBA) (26)	Quantitative non-randomized; post only; cross-sectional	6 workplaces; (75%); Purposive sample of workplaces. 2 workplaces unsuccessful in achieving HBA compared to employees at 4 successful workplaces	Not reported	Questionnaire assessed: attitudes and perception of diet; influences on food choice; knowledge about healthier eating; meals eaten at work/home, food intake at work/home, demographic data.	*Questionnaires:* Wilcoxon signed rank test compared responses between employees at unsuccessful workplaces and successful workplaces; analyses adjusted for employee age, gender, ethnicity, social class, body mass index	Leicestershire Health	High
Holdsworth; 2004; Heartbeat Award scheme (HBA) (27)	Quantitative descriptive; pre-post; cross-sectional	6 workplaces; (75%); Purposive sample of workplaces	Not reported	Employee survey assessed employee dietary practices	Comparison group: 2 workplaces unsuccessful in achieving HBA compared to 4 successful workplaces; Odds ratios and logistic regression used to assess impact of HBA participation on healthiness of purchases; age, gender, ethnicity, social class, BMI	Leicestershire Health	Medium
Jaskiewicz; 2013; Healthy HotSpot (28)	Quantitative descriptive; post only; cross-sectional	25 stores; Convenience sample of stores who participated in Healthy HotSpot initiative	Not relevant	Process evaluation of relevant program records, including staff emails, assessment forms, training records,	Not reported	Centers for Disease Control and Prevention	High

First author surname; Year of publication; Intervention "name"	Study design; Study timing; Length of pre-intervention; intervention; follow-up	Retail outlet sample size (Response rate if relevant and reported); recruitment method if reported	Individual respondent sample size (Response rate if relevant and reported); recruitment method if reported	Data source/s used- Measurement instrument/ tool	Analytical methods	Funding	Study quality
				outreach reports, and other documentation.			
Lynch; 2021; Good Food Corner Stores (29)	Qualitative; post only; cross-sectional	Purposive sampling of retailers who participated in pilot intervention (n=5). Random sampling of retailers who did not participate in pilot intervention (n=3).	Not relevant	Semi-structured interviews. Questions based on previous healthy corner store literature and Diffusion of Innovations Theory	*Interviews*: Thematic analysis	Canada Research Chairs, Ottawa Public Health,<br><br>Canadian Institutes of Health Research	High
Macaskill; 2003; Eat Smart! Ontario's Healthy Restaurant Program (30)	Quantitative descriptive; post only; cross-sectional	319 retailers (74%); Random sampling	Not relevant	Survey of participating retailers	*Surveys:* Descriptive analysis	Canadian Foundation for Dietetic Research	Low
MacAuslan; 1995; Heartbeat Award (HBA) (31)	Qualitative; post only; cross-sectional	16 retailers; Purposive sample of retailers in busy streets	Not relevant	Retailer questionnaire assessed retailer knowledge and understanding	*Questionnaire:* Qualitative summaries	Not reported	Low
Martinez-Donate; 2015; Waupaca Eating Smart (32)	Randomized controlled trial; pre-post; 10-month follow-up	7 restaurants, 2 grocery stores; All participating retailers participated in study	*Restaurants:*<br>152 pre-intervention, 151 post-implementation; Convenience sample<br><br>*Stores:*<br>96 pre-intervention, 203 post-implementation; Convenience sample	NEMS survey to assess changes to food environment<br><br>Customer surveys<br><br>Manager surveys<br><br>Direct observation of outlet practices	Comparison group of 7 non-participating restaurants, 2 grocery stores;<br>*NEMS Surveys:* Descriptive statistics.<br>*Customer satisfaction surveys*: linear and logistic regression; adjusted for age, gender, education, local/ visitor, weekday/ weekend, time of day, special event/ holiday.	University of Wisconsin School of Medicine and Public Health<br><br>National Institutes of Health<br><br>National Heart Lung and Blood Institute	Medium
Matwiejczyk; 2007; Start Right - Eat Right (SRER) (33)	Mixed methods; pre-post no control; 2-year pilot study	44 (88%) long day care centres; All long day care centres in the study	Not relevant	Qualitative telephone feedback from 10 LDCs was used to evaluate the overall impact of SRER	Not reported	Australian Government Department of	Low

First author surname; Year of publication; Intervention "name"	Study design; Study timing; Length of pre-intervention; intervention; follow-up	Retail outlet sample size (Response rate if relevant and reported); recruitment method if reported	Individual respondent sample size (Response rate if relevant and reported); recruitment method if reported	Data source/s used- Measurement instrument/ tool	Analytical methods	Funding	Study quality
		area were eligible to participate		and staff capacity to strengthen nutrition practices.<br><br>Nutrition Checklist assessed menu quality.<br><br>Food policies were assessed against 18 essential criteria pre- and post-SRER.<br><br>Food hygiene and safety was assessed against the Australian Food Premises Assessment or the Food Safe Audit.		Health and Ageing	
McDaniel; 2018; Healthy Retail SF (34)	Qualitative; post only; cross-sectional	6 retailers (out of 17 recruited) interested in participating. Response rate 71%; Purposive sampling of retailers who had and had not expressed interest in participating	Not relevant	Semi-structured interviews	*Interviews:* Inductive thematic analysis compared participating retailers to 11 retailers not interested in participating	University of California, Office of the President, Tobacco-Related Disease Research Program	High
Minkler; 2019; Healthy Retail SF (35)	Mixed methods; pre-post.<br>Sales data assessed for 12 months from baseline to follow-up.<br>Store environment assessed 2- and 4-years post-baseline	10 stores; Purposive sampling	>100 residents leaving near Healthy HotSpot stores; Convenience sample	Store level observational data through a modified “Retail Standards for Health and Sustainability” tool Surveys with local residents.	Not reported	University of California, Office of the President, Tobacco-Related Disease Research Program<br><br>The California Endowment	Low

First author surname; Year of publication; Intervention "name"	Study design; Study timing; Length of pre-intervention; intervention; follow-up	Retail outlet sample size (Response rate if relevant and reported); recruitment method if reported	Individual respondent sample size (Response rate if relevant and reported); recruitment method if reported	Data source/s used- Measurement instrument/ tool	Analytical methods	Funding	Study quality
Moran; 2016; The Healthy Hospital Food Initiative (36)	Quantitative descriptive; pre-post; Varied per site between 4 months to 4 years	28 hospital cafeterias and cafes across 16 public hospitals and 24 private hospitals.	Not relevant	Hospitals submitted patient menus and diagrams of vending machines.<br>Environmental scan assessed café and cafeteria offerings.	Chi squared tests	Centers for Disease Control and Prevention	High
Murphy; 1994; Heartbeat Award (HBA Wales) (37)	Mixed-methods; post only; cross sectional	117 retailers (62%); Convenience sampling of participating retailers 22 local authorities (81%); Convenience sampling of participating local authorities	Not relevant	Post-implementation survey	Participating local authorities were compared to non-participating local-authorities (n=7; response rate 70%)	Not reported	Low
Orme; 2011; Food for Life Partnership (38)	Mixed methods; post only; cross-sectional	111 schools; Convenience sample – first schools to enrol in program	111 school program leads.<br>Subsample of 55 schools participated in pupil and parent surveys (n = 4700 pupils and 1080 parents)	Pupil and parent surveys.<br>School-led surveys and interviews.<br>Program documentation (meeting minutes, strategic plans and reports)	*Surveys:* Descriptive statistics. Regression analysis to test associations between key variables.<br>*Documents:* Thematic analysis of text and transcripts.	Big Lottery Fund	High
Paek; 2014; Project FIT (39)	Quantitative descriptive; pre-post; 2 years	4 corner stores; Purposive sampling of stores that had participated in a previous Nutrition Environment Measures Survey in Stores (NEMS-S)	401 and 318 customers surveyed pre- and post-implementation; Small survey booth established at front of stores to recruit	NEMS-S used to rate stores on accessibility of healthy food.<br>Customer surveys assessed awareness, consumption and store visit frequency pre- and post-implementation.	*NEMS-S*: Descriptive statistics<br>*Customer surveys*: Chi-squared and T-tests compared results pre- and post-implementation. Sensitivity analyses (logistic regression) accounted for customer ethnicity, gender, age and frequency of store visits across customer surveys.	BlueCross BlueShield of Michigan<br><br>USDA Supplemental Nutrition Assistance Program<br><br>Michigan Department of Human Services	High

First author surname; Year of publication; Intervention "name"	Study design; Study timing; Length of pre-intervention; intervention; follow-up	Retail outlet sample size (Response rate if relevant and reported); recruitment method if reported	Individual respondent sample size (Response rate if relevant and reported); recruitment method if reported	Data source/s used-Measurement instrument/ tool	Analytical methods	Funding	Study quality
						Grand Rapids Public Schools Nutrition Services	
Paluta; 2019; Fresh Foods Here (40)	Quantitative descriptive; pre-post; cross-sectional; 10 months	8 stores; Purposive sampling of participating stores	383 customers; Convenience sampling	Store audits: Healthy food availability (inventory), healthy food sales (invoices), store patronage	T-tests compared customer knowledge pre- and post-implementation; store size, stock size.	Not reported	High
Pollard; 2001; Start Right - Eat Right (SRER) (41)	Mixed methods; post only; cross-sectional; data collected at 3 months and 9 months after scheme launch.	134 day-care centres; Recruitment unclear	Not relevant	Telephone interviews conducted with centre coordinators	Not reported	Health Department of Western Australia	Medium
Redelfs; 2021; Eat Well El Paso! (42)	Mixed methods; post only; cross-sectional	1 program coordinator, 1 program recruiter, 1 registered dietitian, participating 26 retailers; purposive sampling	Not relevant	Key informant interviews<br>Store observations<br>Official program documents	*Observations and documents:* Descriptive analysis to describe program uptake and adherence<br>*Interviews:* Thematic analysis to describe barriers and enablers to participation	Healthy Eating & Active Living Initiative of the Paso del Norte Health Foundation	Medium
Rushakoff; 2017; Healthy2Go (43)	Quantitative descriptive; pre-post; 1 year	10 corner stores; Purposive sampling of stores located in a 'food desert';	287 residents in local area at baseline and 281 residents at follow-up; Convenience sampling	Nutrition Environment Measures Survey-Corner Stores (NEMS-CS) (to assess the food environment;<br>Country store inventory logs, storeowner survey, the Community Nutrition Survey (CNS) to assess purchasing and eating patterns.	*NEMS-CS and inventory:* Chi-squared tests and t-tests compared inventory and purchasing pre- and post-implementation.	Centers for Disease Control and Prevention	Medium
Snowdon; 1998; Heartbeat Award (HBA) (44)	Mixed-methods; post only; cross sectional	19 caterers; All eligible caterers included	100 customers from 10 retailers; Convenience sample	Customers: Quantitative surveys	Not reported	Not reported	Low

First author surname; Year of publication; Intervention "name"	Study design; Study timing; Length of pre-intervention; intervention; follow-up	Retail outlet sample size (Response rate if relevant and reported); recruitment method if reported	Individual respondent sample size (Response rate if relevant and reported); recruitment method if reported	Data source/s used- Measurement instrument/ tool	Analytical methods	Funding	Study quality
				Caterers: Qualitative interviews			
Sosa; 2014; ¡Por Vida! (45)	Mixed methods; post only; cross-sectional	4 workplaces; Recruited by the Healthy Restaurant Coalition	7 retailers for key informant interviews 146 customers surveyed	Environmental assessments, customer awareness surveys, key informant interviews	*Customer survey data*: Logistic regression assessed the influence of each personal and environmental characteristic on the likelihood of patrons to choose the ¡Por Vida! menu item *Key informant interviews*: Thematically analysed.	Not reported	Medium
Sosa; 2014; ¡Por Vida! (46)	Quantitative descriptive; post only; cross-sectional	23 restaurants recruited; Random sample of participating restaurants	192 customers; Surveys made available to customers after ordering	Patron awareness surveys- 10 items assessed demographic characteristics; attitudes toward nutrition information; perceived importance of nutrition, taste, and cost; logo visibility; and purchased foods and beverages.	*Customer surveys:* Chi-square test and logistic regression compared responses from customers who saw the ¡Por Vida! logo compared to customers who did not. Analysis adjusted for age, ethnicity, gender	Not reported	High
Warm; 1997; Heartbeat Award (47)	Quantitative descriptive; post only; cross-sectional	380 retailers; Purposive sample of all HBA retailers	Not relevant	Postal questionnaire assessed changes required to achieve award, motivations for enlisting in the award, and current catering practices	*Questionnaire:* Comparison to 306 matched controls without HBA; Chi squared test	Not reported	Low
Zaltz; 2018; ABC Grow Healthy nutrition standards (48)	Quantitative descriptive; post-only; cross-sectional	163 (62%) retailers surveyed	Not relevant	Retailer surveys to identify facilitators of scheme success	*Retailer surveys:* Wilcoxon rank-sum tests, exact Pearson chi-squared tests comparing participating and 84	Robert Wood Johnson Foundation	High

First author surname; Year of publication; Intervention "name"	Study design; Study timing; Length of pre-intervention; intervention; follow-up	Retail outlet sample size (Response rate if relevant and reported); recruitment method if reported	Individual respondent sample size (Response rate if relevant and reported); recruitment method if reported	Data source/s used-Measurement instrument/ tool	Analytical methods	Funding	Study quality
					non-participating retailers.		

Appendix 5: Mixed Methods Appraisal Tool (MMAT) results

First author surname; Year of publication	S1	S2	1.2	1.2	1.3	1.4	1.5	2.1	2.2	2.3	2.4	2.5	3.1	3.2	3.3	3.4	3.5	4.1	4.2	4.3	4.4	4.5	5.1	5.2	5.3	5.4	5.5	Overall quality
Adams; 2012			Y	Y	Y	Y	Y											Y	?	Y	Y	Y	Y	Y	Y	Y	Y	100%
Andreyeva; 2018																		Y	Y	Y	N	Y						80%
Andreyeva; 2022																		Y	Y	Y	N	Y						80%
Bagwell; 2013			Y	Y	Y	Y	Y											Y	?	?	?	?	Y	Y	Y	Y	Y	73%
Bell; 2012																		Y	Y	Y	?	Y						80%
Bell; 2015																		Y	?	Y	Y	Y						80%
Biediger-Friedman; 2014																		?	?	Y	?	?						20%
Boelsen-Robinson; 2020			Y	Y	Y	Y	Y																					100%
Brown; 2017			Y	Y	Y	Y	Y											Y	?	?	?	?	Y	N	N	N	N	47%
Dannefer; 2012																		Y	?	Y	?	Y						60%
Dawson; 2006			N	N	Y	Y	Y											Y	?	Y	N	?	Y	Y	Y	N	N	53%
DeWeese; 2016	Y	Y																Y	?	Y	?	Y						60%
Dombrowski; 2019	Y	Y	Y	Y	Y	Y	Y											Y	?	Y	?	?	Y	N	N	N	Y	60%
Dwivedi; 1999	Y	Y																Y	?	Y	?	N						40%
Dwyer; 2004	Y	Y	Y	Y	?	N	Y																					60%
Economos; 2009	Y	Y	Y	Y	Y	Y	Y											Y	Y	Y	Y	N	Y	Y	Y	Y	Y	93%
Escaron; 2016	Y	Y																Y	?	Y	?	?						40%
Fitzpatrick; 1997	Y	Y	Y	Y	N	N	Y											Y	N	Y	Y	Y	Y	Y	Y	N	Y	73%
Gase; 2015	Y	Y																Y	Y	Y	Y	N						80%
Gase; 2016	Y	Y	Y	Y	N	N	Y											Y	?	Y	N	Y	Y	Y	Y	Y	Y	73%
Gray; 2015	Y	Y	Y	Y	N	N	N																					40%
Gray; 2017	Y	Y	Y	Y	Y	Y	Y																					100%
Green; 1993	Y	Y																Y	?	Y	?	N						40%
Holdsworth; 1997	Y	Y																Y	?	Y	?	Y						60%

**First author surname; Year of publication**	**S1**	**S2**	**1.2**	**1.2**	**1.3**	**1.4**	**1.5**	**2.1**	**2.2**	**2.3**	**2.4**	**2.5**	**3.1**	**3.2**	**3.3**	**3.4**	**3.5**	**4.1**	**4.2**	**4.3**	**4.4**	**4.5**	**5.1**	**5.2**	**5.3**	**5.4**	**5.5**	**Overall quality**
Holdsworth; 1999	Y	Y																Y	Y	Y	Y	N						80%
Holdsworth; 2000	Y	Y											Y	Y	Y	?	Y											80%
Holdsworth; 2004	Y	Y																Y	?	Y	N	Y						60%
Jaskiewicz; 2013	Y	Y																Y	Y	Y	Y	N						80%
Lynch; 2021	Y	Y	Y	Y	Y	Y	Y																					100%
Macaskill; 2003	Y	Y																Y	?	Y	?	N						40%
MacAuslan; 1995	Y	Y	Y	?	?	?	?																					20%
Martinez-Donate; 2015	Y	Y						?	Y	Y	?	Y																60%
Matwiejczyk; 2007	Y	Y	Y	Y	?	N	Y											Y	?	Y	N	N	Y	Y	N	N	N	47%
McDaniel; 2018	Y	Y	Y	Y	Y	Y	Y																					100%
Minkler; 2019	Y	Y																Y	?	Y	?	N						40%
Moran; 2016	Y	Y																Y	Y	Y	Y	Y						100%
Murphy; 1994	Y	Y	?	?	?	Y	?											Y	?	N	?	?	N	Y	Y	N	N	27%
Orme; 2011	Y	Y	Y	Y	Y	Y	Y											Y	?	Y	?	Y	Y	Y	Y	Y	Y	87%
Paek; 2014	Y	Y																Y	Y	Y	Y	Y						100%
Paluta; 2019	Y	Y																Y	Y	Y	?	Y						80%
Pollard; 2001	Y	Y	Y	Y	?	N	Y											Y	?	Y	?	N	Y	Y	Y	Y	Y	67%
Redelfs; 2021	Y	Y	Y	Y	?	?	Y											Y	Y	Y	Y	Y	Y	N	Y	?	Y	73%
Rushakoff; 2017	Y	Y																Y	?	Y	?	Y						60%
Snowdon; 1998	Y	Y	Y	?	?	N	N											Y	Y	N	Y	?	Y	N	N	N	N	27%
Sosa; 2014; Health Behavior and Policy Review	Y	Y	Y	Y	Y	Y	Y											Y	?	Y	Y	Y	Y	N	N	N	Y	73%
Sosa; 2014; Health	Y	Y																Y	?	Y	Y	Y						80%

**First author surname; Year of publication**	**S1**	**S2**	**1.2**	**1.2**	**1.3**	**1.4**	**1.5**	**2.1**	**2.2**	**2.3**	**2.4**	**2.5**	**3.1**	**3.2**	**3.3**	**3.4**	**3.5**	**4.1**	**4.2**	**4.3**	**4.4**	**4.5**	**5.1**	**5.2**	**5.3**	**5.4**	**5.5**	**Overall quality**
Promotion Practice																												
Warm; 1997	Y	Y	N	N	N	N	N											Y	?	Y	?	Y	N	N	N	N	N	20%
Zaltz; 2018	Y	Y																Y	Y	Y	N	Y						80%

Appendix 6: Accreditation scheme characteristics

Name of scheme, target outlet type, country/city, and population characteristics	Primary health goals of scheme	Governance, Compliance monitoring	Rationale for scheme criteria	Food environment scheme criteria (7Ps)	Recruitment strategy	Implementation responsibility and support
Restaurants						
Choose Health LA Restaurants (19, 20)<br><br>Restaurants<br><br>Los Angeles, USA	To promote the availability of healthier menu options. To reduce portion sizes and increase availability of healthy children's meals in restaurants.	*Governance:* The Choose Health LA Restaurants program is a partnership between the Los Angeles County Department of Public Health and local retail restaurants.<br><br>Each application is reviewed and then approved by staff with expertise in the program's administration.<br><br>*Monitoring:* Coordinated through The Los Angeles County Department of Public Health.	Criteria iteratively developed based on formative research	*Product:*<br>Within children's menus: Include one serving of fruits or vegetables per meal, limit to <50% fried foods, only include healthy beverages<br>*Process:* Offer a proportion of menu items in a reduced portion size.<br>*Place:* Free drinking water must be available.<br>*Promotion:* Increase customer awareness of the program via in-store community engagement, promotions, and media campaigns	The DPH sent a letter to all licensed retail restaurants in Los Angeles County. Program staff conducted outreach with potential restaurants, responded to inquiries from interested operators.	To participate, restaurants must submit an application demonstrating menus that adhere to the program participation criteria.<br><br>Technical assistance is available to assist with the application process and menu changes.<br><br>Participating restaurants receive an identifying window decal and in-store promotional materials and are listed on the Choose Health LA website.
Eat Smart! Ontario's Healthy Restaurant Program (30)<br><br>Restaurants<br><br>Ontario, Canada	Not clear	*Governance:* Standardised provincial health promotion program that public health units can adopt.<br><br>*Monitoring:* Not clear	Not clear	*Product:* Meet standards in nutrition and food safety. Standards not available online.<br>*Place (non-food):* Provide non-smoking seating	Not clear	Implementation responsibility unclear. Consumers are made aware of the program through local dining guides, websites, and provided marketing materials.
Fresh choice (18)<br><br>Restaurants<br><br>Vancouver, Canada	To increase availability and accessibility of good-tasting, lower-fat menu items in restaurants.	*Governance:* Developed as a partnership among the Vancouver Health Department, the Restaurant and Foodservices Association of Greater Vancouver,	Not clear.<br><br>Fat-content of products is specifically targeted. Goal is to promote foods with 'little or no-fat'.	*Product:* Prepare Fresh Choices items including soups, salads and desserts: with little/no fat added, soups with clear broth or vegetable-based, desserts with had a fruit	Participation was voluntary. Recruitment strategy not described.	Chefs, in consultation with dietitians, identified or modified menu items that met the lower-fat criteria of the Fresh Choice program.

Name of scheme, target outlet type, country/city, and population characteristics	Primary health goals of scheme	Governance, Compliance monitoring	Rationale for scheme criteria	Food environment scheme criteria (7Ps)	Recruitment strategy	Implementation responsibility and support
	To provide consumers with information to make informed choices.	and the British Columbia Chefs' Association.<br><br>*Monitoring:* Not clear		or baked product as a major ingredient<br>*Process:* Make smaller portion sizes available<br>*Promotion:* Market Fresh Choices<br>*People:* Offer training for staff		Health Department Dietitians provided support with menu analysis and creation, staff training, and program promotion.
Healthier Catering Commitment (HCC) (4, 8)<br><br>Fast food and other restaurants<br><br>London boroughs. London, United Kingdom	To encourage businesses in the catering trade to reduce the levels of saturated fat, salt and sugar in foods, offer healthier options and/or smaller portions, and adopt healthier cooking and preparation practices by using the 'small changes make a big difference' principle.	*Governance:* Developed by the Greater London Authority, the Chartered Institute of Environmental Health, and the Association of London Environmental Health Managers in 2012.<br><br>*Monitoring:* Environmental Health Officers responsible for monitoring and enforcement.	The Healthier Catering Commitment scheme follows on from a range of initiatives targeted at out-of-home caterers.	*Product:*<br>1.Eliminate choice of some unhealthy products, notably unhealthy oils and salt.<br>2. Enable choice of healthier products. In some cases the healthier choice must be the default<br>*Process:* Guide choice by changing default policy (such as customers adding own salt)<br>*People:* Healthy eating is promoted by staff	Environmental Health Officers recruited food businesses and supported them in meeting specific criteria related to the healthiness of their food and drink offerings.	Environmental Health Officers delivered Healthier Catering Commitment with the support of the public health team as part of their normal duties. Alternatively, delivery was contracted to an external organisation with support from Environmental Health Officers.
Heart Smart Restaurant Program (14, 23)<br><br>Restaurants<br><br>Canada (evaluations conducted in Ottawa-Carleton, and Saskatoon and Regina)	To make more healthful food choices more readily available in table-service restaurants and to encourage restaurant patrons to make those choices.	*Governance:* Scheme created by the Heart and Stroke Foundation of Canada.<br><br>*Monitoring:* Volunteers administered a monitoring survey in some cities where the program was implemented.	Guidelines were adapted from the original Heart and Stroke Heart Smart program.	*Product:* Offer lower fat / higher fibre food options (meat, dairy, grains, dressing and sauces, desserts). Use healthier (vegetable) as opposed to less healthier oils.<br>*Promotion:* Promotional materials used<br>*Process:* Food is prepared without added sodium. Food is boiled or roasted rather than fried. Visible fat is removed. Sauces and dressings are served	Not clear	Restaurant implementation with volunteer support. Volunteers maintained retailer engagement, provided suggestions on how to meet program criteria, and provided promotional materials.

Name of scheme, target outlet type, country/city, and population characteristics	Primary health goals of scheme	Governance, Compliance monitoring	Rationale for scheme criteria	Food environment scheme criteria (7Ps)	Recruitment strategy	Implementation responsibility and support
				on the side and lower fat options are used.		
Savvy Diner (9)<br><br>Independent restaurants (locally owned, not chains or franchises, or with fewer than 4 locations)<br><br>Toronto, Canada	To make nutrition information more available and visible to consumers	*Governance:* Toronto Public Health<br><br>*Monitoring:* Researcher-led	Pilot components were designed after consulting with jurisdictions implementing similar initiatives and were refined with feedback from interested restaurants and industry associations.	*Product:* Create standardised recipes and conduct nutrient analysis on these products<br>*Process:* Provide nutrient/calorie information	All independent restaurants in Toronto were invited by mail to complete an online survey which assessed readiness for menu labelling and asked about interest in participating in a pilot program.	Public Health Dietitians trained in recipe standardization and nutrient analysis each dietitian supported 2–3 restaurants.<br><br>The following supports were offered:<br>• Orientation through an on-site restaurant visit<br>• Subscription to online nutrient analysis software<br>• Confidential on- and off-site consultation from a dietitian<br>• “Savvy Diner Approved” designation and promotional materials<br>• Recognition and promotion of the restaurant via traditional and social media platforms.
Shape Up Somerville: Eat Smart, Play Hard (16)<br><br>Restaurants<br><br>Somerville, Massachusetts, USA. Focus on families and children.	To support a healthy environment within the community by working with local restaurants, especially restaurants frequented by families, to increase the availability of healthful alternatives	Governance and monitoring not clear.	National School Lunch Program regulations	*Product:* Offer smaller portion sizes, fruits and/or vegetables as a side dish or entree, low-fat/no-fat dairy products<br>*Promotion:* Highlight healthier options on menus and display a Shape Up Somerville seal on the restaurant door/window	Restaurants were prioritized by type and potential for change. Family-friendly sit-down restaurants were given highest priority, followed by delicatessens and sandwich shops. Staff first attempted to contact retailers by telephone, then mail, and finally in-person visits. Recruitment	Approved restaurants received:<br>1-inch stickers that could be placed on existing menus, boards or signs and were given assistance in designing menu inserts; a 4-inch window decal; and laminated signs and table tents listing the “Shape Up Approved” criteria.

Name of scheme, target outlet type, country/city, and population characteristics	Primary health goals of scheme	Governance, Compliance monitoring	Rationale for scheme criteria	Food environment scheme criteria (7Ps)	Recruitment strategy	Implementation responsibility and support
	and smaller portions of food.				kits included an information guide, Shape Up Somerville contact information, newsletter, a letter of agreement, media articles, and a list of participating restaurants.	
¡Por Vida! Initiative (7, 45)<br><br>Restaurants<br><br>San Antonio, Bexar County, Texas, USA<br><br>High proportion Hispanic.	To highlight healthier options for restaurant patrons.	*Governance:* San Antonio's Healthy Restaurant Coalition oversaw the initiative - made up of San Antonio Metropolitan Health District, the San Antonio Restaurant Association, and the San Antonio Dietetic Association.<br><br>*Monitoring:* Registered dietitians from the Nutrition Criteria Committee evaluated menu items for fit to the criteria.	2005 Dietary Guidelines for Americans (balanced diet consisting of fruits, vegetables, whole grains, low-fat dairy, and lean protein)	*Product:* Provide at least one healthier menu item meeting nutrient criteria:<br>An adult meal: <700kcal, <23g total fat, <8g saturated fat, <0.5g trans-fat, <750mg sodium.<br>A single item: <300cal, <10g total fat, <3.5g saturated fat, <0.5g trans-fat, and <325mg sodium.<br>A side item: <200cal, <7g total fat, <2g saturated fat, 0.5g trans-fat, and 215mg sodium.<br>*Promotion:* Promote healthier menu items to customers through logo placement at entryways and on menus; competitions to create the next ¡Por Vida! Recipe, and ¡Por Vida! advertising on flyers and table tents in dining areas.	Initiative was voluntary. Restaurants were invited to participate if they had or were willing to modify a menu item to meet criteria.<br>The Restaurant Selection and Implementation committee was comprised of representatives from Metro Health and San Antonio Restaurant Association.	Participating restaurants received promotional materials and recognition on the ¡Por Vida! Website.<br>Restaurants engaged in a 4-to-6-month process with a registered dietitian. The dietitian analysed the menu and developed options to modify recipes.
Eat Well El Paso! (EWEP) (42)<br><br>Restaurants	Increase healthy food choices for adults and children by improving food offerings, using healthier cooking	*Governance:* El Paso Public Health Department<br><br>*Monitoring:* Not described	-USDA School Lunch program<br>-USDA dietary guidelines for children<br>-Dietary Recommendations for	*Product:* Meals must include protein, starch, vegetable or fruit. Appetizers, side dishes or deserts should include wholegrains. Nutrition	Not described	Registered dietitians provided support with altering menus.<br><br>EWEP staff provided newly designed menus,

Name of scheme, target outlet type, country/city, and population characteristics	Primary health goals of scheme	Governance, Compliance monitoring	Rationale for scheme criteria	Food environment scheme criteria (7Ps)	Recruitment strategy	Implementation responsibility and support
El Paso, Texas, USA	methods, and offering healthier beverage options.		Healthy Children of the American Heart Association<br>-National School Lunch Program for K-5<br>-American Heart Association Heart Check Certification Program for Heart Healthy Meals<br>-2010 Dietary Guidelines for Americans	standards set for meals, mains and sides/ appetizers/ deserts, for adults and children. Limits exist for calories, fat, saturated fat, sodium. Minimum levels exist for calcium, vitamin C, potassium, fibre. No SSBs to be provided with meals		trained restaurant staff, and promoted participating restaurants
Corner stores						
Change4Life (1)<br><br>Change4Life convenience store intervention<br><br>Socio-economically deprived, urban areas with poor existing retail access to FFV in northeast England.	Improve provision of FFV in convenience stores	*Governance:* Department of Health civil servants provided strategic leadership for the intervention. A national steering group consisted of the Department of Health team, the project delivery team, and national representatives of each of the symbol groups involved.<br><br>*Monitoring:* 'Fresh Food Champion' responsible for compliance. A regional steering group was also established consisting of representatives of the Department of Health leadership team, the project delivery team, regional symbol group managers, the regional	No pre-existing criteria. Specific goal was to promote and increase availability of FFV.	Differing changes made in 'demonstration' and 'roll-out' stores. In Demonstration stores:<br>*Product:* Increase provision (greater quantity) of FFV<br>*Place:* Layout store to promote FFV.<br>*Promotion:* Display Change4Life branded materials. Launch leaflet in Healthy Start mailings, sent to households within a 1-mile radius;<br>*Process:* Provide in-store sampling of FFV	Symbol group stores have common brand identity, and access wholesale goods via regional managers. Stores were selected for inclusion by consultation between the project delivery team and regional symbol group managers.	Implementation co-ordinated by intervention delivery team. 'Fresh Food Champion' responsible for in-store implementation.<br><br>Intervention delivery team supplied branding materials, intervention support, staff training materials, and appointed an existing staff member as 'Fresh Food Champion' to oversee compliance. Department of Health provided 50% of costs for a new chill cabinet for FFV.<br><br>Intervention cost £5100 per demonstration store. £300 for roll-out store.

Name of scheme, target outlet type, country/city, and population characteristics	Primary health goals of scheme	Governance, Compliance monitoring	Rationale for scheme criteria	Food environment scheme criteria (7Ps)	Recruitment strategy	Implementation responsibility and support
		obesity lead, and local health workers.				Stores were supported to link in with other Change4Life initiatives, including Cook4Life and Breakfast4Life.
Healthy HotSpot (HH) initiative (13, 28).<br><br>Corner stores<br><br>Low-income, urban communities in suburban Illinois, USA	To transform corner stores into a health-promoting resource for the community and increase sales of healthful foods in corner stores.	*Governance:* Led by the Cook County Department of Public Health and the Public Health Institute of Metropolitan Chicago. Local community institutions became integral partners in the initiative because of detailed community knowledge and local presence.<br>*Monitoring:* US$200,000 budget for store incentive payments, store equipment, and community institution resources.	Not clear	*Product:* Add 6 new foods: 1 fresh fruit, 1 fresh vegetable, and 4 foods chosen from low-fat dairy, lean proteins, canned or frozen fruits and vegetables, or whole grain categories<br>*Promotion:* Market heathy food (second 'conversion' phase of scheme rollout only)<br>*People:* Provide workshops and webinars provided guidance on community outreach, marketing and pricing for staff.<br>Corner stores who complied received enhanced community outreach and engagement by the local organisation (*Partnership*) and<br>a plan for healthy product and equipment placement (*Place*).	HH staff contacted community institutions to request assistance with recruitment. Community institutions were responsible for identifying and recruiting local corner stores into the initiative.	Retailers were responsible for adding new products. Stores that successfully added the new foods received US$250 and were invited to participate in the second phase (conversion).<br><br>HH staff visited stores to identify equipment needs. The conversion phase provided stores with new equipment, marketing materials, enhanced community outreach and engagement, and an additional US$250.
Fresh Foods Here (40)<br><br>Corner stores	To improve access to healthy foods and beverages in small, independent stores located in low-income	*Governance:* Not clear<br><br>*Monitoring:* Scheme coordinators conducted store inventories and collected POS invoices,	Foods had to meet 2 of the following criteria to be considered healthy: low sodium, low fat, and/or low sugar.	*Product:* Increase availability of healthy foods.<br>*Promotion:* Market healthy food.	Recruitment strategy not clear.<br><br>Incentives were structured to reward engagement from retailers	Retailer-led implementation.<br><br>Stores were provided with technical and marketing assistance and assistance

Name of scheme, target outlet type, country/city, and population characteristics	Primary health goals of scheme	Governance, Compliance monitoring	Rationale for scheme criteria	Food environment scheme criteria (7Ps)	Recruitment strategy	Implementation responsibility and support
Franklin County, Ohio<br><br>Areas where low-income households, rely on food stores located in their local food environments	neighbourhoods with limited healthy food access throughout Columbus.<br><br>To achieve the primary health aim in ways that will be profitable for business-owners	and trained volunteers conducted customer surveys and rapid market assessments.			and to sustain healthy food retailing by diversifying stores' business models and attracting customers to stores with healthy food options	with physical store improvements
Healthy Bodegas Initiative (10)<br><br>Corner stores/bodegas<br><br>South Bronx and East New York, USA<br><br>Low income, high rates of overweight, obesity and diabetes	(i) To support environmental change in corner stores by offering simple, low-cost, and effective mechanisms for selling and promoting healthier items<br><br>(ii) To work with community organizations and residents to support the purchase of healthier foods to ensure sustainable inventory change	*Governance:* Coordinated by the New York City Department of Health and Mental Hygiene and funded by the New York City Center for Economic Opportunity.<br><br>*Monitoring:* Outreach staff visited stores 10 times over 5 months to assess progress toward meeting the criteria and to identify areas for improvement.	Criteria developed from a range of sources, including nutritionists' input, customers' input, and trial and error from previous Healthy Bodegas Initiatives.<br><br>Criteria were also based on health department priorities including increasing access to fresh produce and limiting sugary drink consumption.	A total of 16 criteria available to be achieved.<br>Level 1: meet 7 criteria<br>Level 2: meet 13 criteria<br>Level 3: meet 16 criteria<br>Criteria related to:<br>*Product:* Improve healthy/ unhealthy product stocking in stores (based on stocking of different quantities of fruit, vegetable, wholegrain varieties).<br>*Place:* Promote healthier products via displays<br>*Promotion:* Display promotional posters promoting healthy eating<br>*People:* Staff participate in 20-minute store training program	Bodegas received incentives as they advanced levels, including produce display crates and reusable shopping bags to offer to customers who purchased fresh produce. Stores also received promotional materials to market their healthier products to customers. Program staff linked bodegas with community groups to support changes.	Scheme staff provided technical assistance, such as rearranging the store to increase the visibility of healthy items, posting materials promoting healthier items, and assisting with applications for microfinancing and permits.
Healthy2Go (43)<br><br>Corner stores<br><br>Four counties in the Cumberland Valley area; Bell, Clay,	To improve the availability of healthy food options in a rural, low quality food environment setting	*Governance:* Program designed by Spread the Health Appalachia.<br><br>*Monitoring:* Spread the Health Appalachia staff	Healthy food categories were established based on a review of corner store transformation programs nationally and with input from The Food Trust and local nutritionists.	Stores could meet criteria across 3 phases to qualify for award. Criteria covered:<br>*Product:* Include more healthy foods in store inventories	All stores located in a food desert or a food poor census tract qualified. Stores were provided information on program. Owners self-selected to participate, committing to	Retailers and Spread the Health Appalachia staff shared implementation. Stores were provided with promotional materials. Taste testings were put on and staff training

Name of scheme, target outlet type, country/city, and population characteristics	Primary health goals of scheme	Governance, Compliance monitoring	Rationale for scheme criteria	Food environment scheme criteria (7Ps)	Recruitment strategy	Implementation responsibility and support
Jackson, and Knox, in south-eastern Kentucky, USA. Areas were considered 'food deserts'.		conducted all monitoring surveys.		*People:* Train store owners and staff<br>*Promotion:* Use point-of-purchase materials, and host community events<br><br>Specific criteria or phases were not published	meet criteria in each of the 3 program phases, to participate in technical assistance and training programs.	programs and reading materials were available. Additional equipment was also installed
Healthy Retail SF (34, 35)<br><br>Corner stores<br><br>Food insecure, ethnically diverse neighbourhoods (notably Tenderloin), San Francisco, USA.	To improve healthy food access while decreasing availability and advertising of tobacco and other unhealthy products in food insecure neighbourhoods.<br><br>To increase the financial viability of local corner stores that become healthy retailers	*Governance:* Led by the Tenderloin Healthy Corner Store Coalition<br><br>*Monitoring:* The Tenderloin Healthy Corner Store Coalition employed and trained 8 food justice leaders to manage data collection for monitoring/evaluation purposes.	Healthy foods defined as fresh fruits and vegetables, wholegrains, low-fat dairy and lean proteins.	*Product:* Increase space for fresh produce, whole grains, lean proteins, and low-fat dairy products to 35%.<br>*Promotion:* Use promotional events, marketing materials, and taste testing and demos<br>*Place:* The physical environment of the store and product placement<br>*Process:* Evaluate and redesign business processes<br>*Partnership:* There are several partnerships with local community organisations	Participating corner stores in Tenderloin were selected following a formal application process. Factors such as store location, size, and level of commitment were key considerations in the selection process.	The Tenderloin Healthy Corner Store Coalition and the San Francisco Department of Public Health, in partnership with participating stores and Sutti architects, installed equipment and signage.<br><br>US$24,000 support provided/store ($20,000 for equipment and consulting; $1000 POS system; $3000 for equipment).<br>Additional support included:<br>-store redesign<br>-art to replace unhealthy advertisements<br>-technical assistance<br>-marketing research<br>-promotional events
FIT Store (part of Project FIT) (39)<br><br>Corner stores	To make healthy foods more available and affordable in low-income, urban, and ethnically diverse	*Governance:* Collaboration among a number of universities, school organisations, NGOs and community partners	No framework for nutritional classification provided	*Product:* Increase availability of healthy products<br>*Promotion:* Display nutrition information and marketing materials	Recruited from those who had participated in a NEMS-S survey of stores in 4 neighbourhoods as part of the Project FIT program. Survey	Retailer-led implementation.<br>Stores were provided with:<br>(i) Small grants for equipment

**Name of scheme, target outlet type, country/city, and population characteristics**	**Primary health goals of scheme**	**Governance, Compliance monitoring**	**Rationale for scheme criteria**	**Food environment scheme criteria (7Ps)**	**Recruitment strategy**	**Implementation responsibility and support**
Low-Income, Urban, and Ethnically Diverse Neighbourhoods in Grand Rapids, Michigan, USA	neighbourhoods in Grand Rapids, Michigan.	*Monitoring:* Researcher-led surveys.		*Price:* Make healthy products more affordable<br>*People:* Train staff in healthy food training	participants were encouraged to apply for a pilot healthy corner store program.	(ii) Assistance with identifying sources of fresh/healthy foods<br>(iii) Training for store owners/ managers<br>(iv) Help with marketing healthy foods<br>(v) Nutrition information materials to place on site.
United States Department of Agriculture's Special Supplemental Nutrition Program for Women, Infants, and Children (WIC) AND Supplemental Nutrition Assistance Program (SNAP) (12)<br><br>Corner stores<br><br>Four cities (Camden, Newark, New Brunswick, and Trenton) in New Jersey, USA.<br><br>Focus on low income and high-minority communities.<br><br>Some focus on Women, Infants,	To increase the availability of healthy foods in corner stores.<br><br>Improve diets and health outcomes for women children and infants (WIC) and all groups (SNAP).	*Governance:* United States Department of Agriculture. Stores are required to apply for and be awarded state-administered certification.<br><br>*Monitoring:* Not clear	To provide support to low-income households for food purchasing.	*Product:* Increase availability of healthy products.<br>SNAP vendors: Sell 3 varieties of foods in all four staple food groups: meat, poultry, or fish; bread/cereal; fruits and vegetables; dairy.<br>WIC vendors: Sell low-fat milk; unsweetened juice; fresh, frozen or canned fruits and vegetables; wholegrains.	Stores that wish to accept SNAP or WIC vouchers must stock the required products.	Retailer-led implementation.<br><br>SNAP and WIC receive federal funding, but funding is not available to specific stores (except through the receipt of vouchers).

Name of scheme, target outlet type, country/city, and population characteristics	Primary health goals of scheme	Governance, Compliance monitoring	Rationale for scheme criteria	Food environment scheme criteria (7Ps)	Recruitment strategy	Implementation responsibility and support
and Children (WIC).						
Good Food Corner Stores (GFCS) (29)<br><br>Corner stores<br><br>Ottawa, Ontario, Canada<br><br>Focus on low-income neighbourhoods without access to a major grocery retailer	Promote, and make it easier for residents to purchase, fresh fruits and vegetables	*Governance:* Public Health Unit of Ottawa, Ontario, Canada<br><br>*Monitoring*: Public health unit staff conducted site visits	To promote fresh fruit and vegetables, alongside other healthy products.	*Product:* Stores must offer at least 3 types of vegetables, 3 types of fruit, 2 types of wholegrain, 2 lower fat milk options, 2 meat alternatives<br>*Promote:* Store must display GFCS marketing materials	Purposive sampling for pilot study.	Public health unit provided branding, promotion, resources (including baskets and shelving materials for displaying FFV), and links to community support.
Schools and childcare centres						
ABC Grow Healthy nutrition standards (48)<br><br>Early care and education (ECE) centres<br><br>Low-income ECE centres in South Carolina, USA	Grow Healthy sets forth policies and practices to increase children's consumption of healthy foods and time spent being physically active while in childcare.	*Governance:* Not clear<br><br>*Monitoring:* Not clear	Not clear	*Product:* Limit unhealthy foods availability, and provide minimum quantities of healthy foods	Not clear	Not clear
Start Right - Eat Right (SRER) (5, 6, 33, 41)<br><br>Long Day Care Centres<br><br>Food outlets in Long Day Care	To increase long day care centre capacity to provide safe, healthy food choices and a positive eating environment for children	*Governance*<br>South Australia: State government award scheme<br><br>Western Australia: The 12-member WA Food Service Childcare Advisory Group with	Children's daily nutrition requirements as recommended by Australian state health departments.	*Product:* Menu must provide at least 50% of children's daily nutrition<br>*People:* Train all staff in food hygiene and safety<br>*Place:* Provide a supportive eating environment for children	Not reported	South Australia: Centre-implementation.<br><br>Western Australia: The Gowrie Inc., a national childcare training and resource organization, was contracted to

Name of scheme, target outlet type, country/city, and population characteristics	Primary health goals of scheme	Governance, Compliance monitoring	Rationale for scheme criteria	Food environment scheme criteria (7Ps)	Recruitment strategy	Implementation responsibility and support
Centres in Metropolitan Adelaide, South Australia, and in Western Australia		representatives from the childcare industry and government<br><br>*Monitoring:* Auditing was conducted by the local government environmental health officer. A local nutritionist or SRER staff member conducted site visits for monitoring of staff behaviour and practices.		*Processes:* Introduce a food policy		implement the scheme state-wide.<br><br>Both states: staff training was offered.
USDA Child and Adult Care Food Program (CACFP) (2, 3)<br><br>Meal provision services in child and adult care settings.<br><br>Child and adult care settings in the USA.<br><br>All non-profit childcare facilities are eligible to participate, regardless of family income for the enrolled children. For-profit facilities are eligible to participate if at least 25% of children	To support childcare nutrition.<br><br>To financially support adult and childcare centres in supporting nutrition.	*Governance:* USDA's Food and Nutrition Service (FNS) administers CACFP through grants to states. The program is administered within states by the state educational agency health or social services department.<br><br>*Monitoring:* State Department of Education.	Dietary Guidelines for Americans (DGAs)	*Product:* Limits on juice and added sugar, including more whole grains, introducing fruit and vegetables as separate meal components.<br>*People:* Staff training criteria.<br>*Process:* Eliminating onsite deep frying as a meal preparation method.<br>*Promotion*: Criteria relating to staff consumption of food in front of children.	Voluntary program. All non-profit childcare facilities are eligible to participate, regardless. For-profit facilities are eligible to participate if at least 25% of children come from low-income families.<br><br>However, in Connecticut, all licensed childcare centres require compliance with the CACFP nutrition standards.	Centre implementation.<br><br>Federally funded program, providing training, guides and other resources. The scheme provides reimbursements for foods served in child and adult care settings that follow set nutrition standards.

Name of scheme, target outlet type, country/city, and population characteristics	Primary health goals of scheme	Governance, Compliance monitoring	Rationale for scheme criteria	Food environment scheme criteria (7Ps)	Recruitment strategy	Implementation responsibility and support
come from low-income families.						
Hospitals						
The Healthy Hospital Food Initiative (36)<br><br>Hospitals<br><br>Hospitals in New York City, USA	To improve the healthfulness of foods and beverages available in New York City hospitals.	*Governance:* The New York City Department of Health and Mental Hygiene oversaw implementation.<br><br>*Monitoring:* Implementation of the standards was monitored through ongoing conversations with hospital staff, site visits, and menu analyses by health department dietitians.	Not clear	*Product:* 4 criteria provided for: (i) Patient meals (sodium content guidelines, 5 servings of fruits/ vegetables per day).<br>(ii) Beverage vending machines (reduce sugary drink portion size, limit sugary drinks to 2 vending machine slots, place water at eye level)<br>(iii) Food vending machines (nutrient guidelines (calories, sodium), and promoting whole foods over grain-based snacks)<br>(iv) Cafes/ cafeterias (limit sodium, sugary drinks, and calorie-dense foods; promote water, fruits, vegetables, whole grains. E.g. ≥50% of all grains in entrees and sandwiches must be whole grains, and ≥75% of all beverages available must be low-calorie (≤25 kcal/8 oz).	The criteria around patient meals and food and beverage vending machines is mandatory for public hospitals. Public hospitals can self-elect to participate in the 4th criteria around cafes/cafeterias.<br><br>Private hospitals were recruited by working with a local hospital association and hospital workers' union. Private hospitals commit to implementing 4 sets of standards.	Hospital staff were responsible for implementation.<br><br>The health department offered technical assistance to hospitals, which included provision of implementation guides, promotional materials, and assistance from 2 full-time registered dietitians.
Workplaces						
Eat Smart! Workplace	To increase the availability of healthier food	*Governance:* Not clear<br><br>*Monitoring:* Not clear	Canada's Food Guide to Healthy Eating	*Product:* Increase availability of healthier products.	Not clear	Implementation responsibility unclear. Hamilton's Public Health

Name of scheme, target outlet type, country/city, and population characteristics	Primary health goals of scheme	Governance, Compliance monitoring	Rationale for scheme criteria	Food environment scheme criteria (7Ps)	Recruitment strategy	Implementation responsibility and support
Cafeteria Program (11)<br><br>Workplaces<br><br>Ontario, Canada	choices in a smoke-free environment, and to promote safe, healthier food choices in the cafeteria.			*Promotion:* Display promotional posters.<br>*Process: P*rovide condiments only upon request.		and Community provided assistance in how to deliver the program. Program was promoted through a range of media.
Multiple settings						
Food For Life Partnership (FFLP) (21, 22, 38)<br><br>Schools, universities, care homes, hospitals.<br><br>United Kingdom	To encourage a healthy, sustainable food culture across communities	*Governance:* Partnership of national charities in England led by the Soil Association, with Garden Organic, Focus on Food, the Health Education Trust and the Royal Society for Public Health<br><br>*Monitoring:* Not clear	*Hospitals:* National nutrition standards<br><br>*Schools:* School Meals Review Panel food-based and nutrient-based standards for school lunches	*Product:* Provide food free from additives and trans fats and aligned with standards.<br>*People:* In schools, staff should act to promote healthy foods to students, and to educate students on healthy foods.<br>*Partnership:* Food culture & community involvement.	Voluntary. Recruitment method unclear	Implementation responsibility unclear. Support provided through the Food for Life Partnership led by the Soil Association
Heartbeat award (HBA) scheme (24-27, 31, 44, 47)<br><br>Workplaces, restaurants, cafés, schools, residential homes, sports centres<br><br>England, United Kingdom	To reduce total fat, sugar, and salt and increase the availability of fibre rich, starchy foods.<br><br>Long-term, to reduce the incidence of coronary heart disease	*Governance:* Initial governance by Health Education Authority which later gave control to local HBA authorities<br><br>*Monitoring:* Caterer completes self-assessment and is then visited by a dietitian. The responsibility for awarding the HBA to establishments lies with environmental health officers from local HBA authority (local units responsible for HBA governance).	The criteria for the Heartbeat Award scheme are set nationally by the Health Education Authority. Healthy choices were defined as low in fat and high in starch and fibre.	*Product:* Provide one third of dishes as healthy choices.<br>*Promotion:* Direct customers to healthier choices using labels.	Not clear	Not clear. Assumed caterer implementation. Dietitian provides some support.

Name of scheme, target outlet type, country/city, and population characteristics	Primary health goals of scheme	Governance, Compliance monitoring	Rationale for scheme criteria	Food environment scheme criteria (7Ps)	Recruitment strategy	Implementation responsibility and support
Heartbeat Award (HBA) administered by Heartbeat Wales (HBW) (37)<br><br>Workplaces, restaurants, cafés, schools, residential homes, sports centres<br><br>Wales, United Kingdom	To develop and evaluate a regional strategy that would contribute to a sustained reduction in coronary heart disease incidence, morbidity and mortality in the general population of Wales.	*Governance:* Developed in partnership with Heartbeat Wales and the institution of Environmental Health Officers<br><br>*Monitoring:* Pilot authorities were responsible for the local management and monitoring of the award. Monitoring was linked to routine food hygiene visits by Environmental Health Officers.	Not clear	*Promotion:* Advertise healthier food options and smoking ban to customers through provision of nutritional education and marketing materials.<br>*Product:* Provide list of healthy items.	Catering establishments identified to have good standards of practice during routine food hygiene visits by Environmental Health Officers were invited to participate.<br><br>Environmental Health Officers distributed leaflets and press releases and spoke with caterers.	Implementation responsibility not clear.<br><br>Caterers who were granted an award received an enhanced range of materials, including, for example, nutrition guidance notes, notes on establishing no-smoking areas, and promotional leaflets.
Waupaca Eating Smart (WES) (17, 32)<br><br>Restaurants and supermarkets<br><br>Midwestern rural communities, USA	To improve the nutrition environment and promote healthy eating in restaurants and food stores of a rural community.	*Governance:* Developed by a university-based academic team and 2 local nutrition and physical activity (NPA) coalitions.<br><br>*Monitoring:*<br>NPA coalition staff visited each outlet unannounced and, using a checklist, recorded which WES materials and other components of the program were present.	Based on a restaurant-based effort to evaluate the extent to which menu offerings meet the Dietary Recommendations for Americans.	*Product:* Offer one or more healthy WES meal<br>*People:* There are criteria relating to staff. understanding WES and promoting WES items<br>*Promotion:* Promote healthier menu items to customers.<br>*Partnership:* Consider sponsoring cooking classes	Initiative was voluntary. Coalition staff actively engaged outlets considered as likely “early adopters” of the intervention and influential opinion leaders, whose example might be followed by additional outlets within the community.	Implementation was shared between the retailer and the NPA coalition. A local registered dietitian in the NPA coalition analysed menu items to identify or create “WES-approved” meals.<br><br>Promotional and point-of-purchase materials were developed by academics and the NPA coalition to identify and promote these meals.

Appendix 7: Outcomes of included accreditation schemes

Scheme name	Uptake and certification rates	Impact on retailer practices	Impact on customer behaviours	Customer perspectives	Retailer perspectives and commercial outcomes
Restaurants					
Choose Health LA Restaurants<br><br>Restaurants	100% (17 of 17 restaurant brands) (19).	12 of the 17 restaurant brands made changes to meet criteria (5 already qualified). 9/17 brands reduced portion sizes (19).<br><br>Among restaurants that made menu changes, the 3 most frequently offered options available in reduced-size portions were salads, sandwiches or burgers, and pasta, pizza, or grain-based main dish (19).<br><br>10/17 brands offered children's meals; 8 of these 10 made changes to their children's meals (19).	Not reported	12% customers reported seeing Choose Health LA Restaurants logo. 56% customers reported that seeing the logo at a restaurant would make them more likely to eat there (43% would not influence their decision, and 1% would make them less likely to eat there). 70% customers correctly identified the meaning of the logo (20).	Not reported
Eat Smart! Ontario's Healthy Restaurant Program<br><br>Restaurants	Not reported	22% of retailers made changes to the food offered. Fewer than 50% of retailers used any of the provided marketing materials correctly (30).	Not reported	Not reported	Retailers mainly chose to participate in the Eat Smart! program to have their restaurants known as clean and healthy places to eat. 98% of retailers planned to continue participating (30).
Fresh choice<br><br>Restaurants	Not reported	Not reported	Customers were not always aware whether they had selected a Fresh Choice (customers could not identify 32% of menu items as Fresh Choice or not) (18).	Customers were significantly more satisfied with lower-fat than regular menu items.<br><br>All customers concluded that there was a need for programs like Fresh Choice in restaurants (18).	Not reported
Healthier Catering Commitment (HCC)	Not reported	**Provision of information:** 26/77 businesses agreed to start promoting healthier eating.	3/12 customers surveyed stated that their reason for choosing the healthier dishes was because it was a 'healthier option' (4).	Not reported	Businesses were concerned that not adding salt would radically alter the taste of the food resulting in a loss of customers.

Scheme name	Uptake and certification rates	Impact on retailer practices	Impact on customer behaviours	Customer perspectives	Retailer perspectives and commercial outcomes
Fast food and other restaurants		**Enabling healthier choices:** 63/77 business reduced the quantity of soft drinks available. 22/77 businesses made lower sugar snacks available. 29/77 ensured that fresh fruit was always available. 46/77 ensured wholegrains were available. **Changing default policy:** 20/77 businesses allowed customers to add their own salt. 10/77 businesses changed to a healthier oil (4).			Businesses reported that most customers had well established preferences and would not be influenced by positioning healthier alternatives at eye level.<br><br>Businesses in less affluent areas were more resistant to offering fruit and wholegrain alternatives and were less likely to believe there was a market for healthier food in their area, compared to businesses operating in more affluent areas (4).<br><br>The only cost to the businesses of adopting a provision of information strategy was printing a separate menu. Most businesses interviewed only fried a small proportion of their menu items, and thus the extra 15% cost of a healthier variety of oil could be relatively easily absorbed (4).
Heart Smart Heart Beat Restaurant Program<br><br>Restaurants	Approx. 68% participation rate in Saskatoon and 56% participation rate in Regina (23).	In Ottawa-Carleton, more than 90% of restauranteurs could provide the healthy food choices. Fewer than 33% used the table tents and menu inserts. Slightly more used the staff poster, and 45% or more used the decal and the certificate (14).<br><br>In Saskatoon, 73% of reported requests for a more healthful alternative were met every time, and 27% sometimes. In Regina, 62% of reported requests for a	In Ottawa-Carleton, 36% of restauranteurs noticed an increase in the number of patrons asking for healthy choices (14).<br><br>In Saskatoon (84.4%) and Regina (82.6%), most customers reported they were not greatly influenced by a restaurant being designated Heart Smart (23).	In Saskatoon, 41.1% of customers were familiar with HSRP, compared to 22.0% in Regina.<br><br>Approximately 50% of customers understood what the program was.<br><br>Most customers (Saskatoon 60.4%, Regina: 65.4%) could not identify correctly how the program worked (23).	92% of restaurants were aware they were participating in the program.<br><br>98% would like to be included if the dining guide was published again (14).

Scheme name	Uptake and certification rates	Impact on retailer practices	Impact on customer behaviours	Customer perspectives	Retailer perspectives and commercial outcomes
		more healthful alternative were met every time and 38% sometimes (23).			
Savvy Diner<br><br>Restaurants	15/22 restaurants completed some aspect of the program; none completed the entire original menu labelling process. 4 restaurants completed all elements of the program according to updated requirements (9).	No restaurants completed the entire original menu labelling process. 9/15 restaurants began recipe standardization and got no further.<br>Two completed nutrient analysis but did not post values for customers to access. 4 restaurants completed all elements of the program according to updated requirements (9).	Not reported	Not reported	Nine restaurant operators participated in structured exit interviews.<br><br>All operators agreed that their experience was positive. They would recommend the program to other restaurants if they: (i) were already serving healthy food, (ii) had personnel and time to dedicate to the program, (iii) saw the benefits of standardizing recipes, and (iv) valued marketing themselves as a healthy restaurant (9).
Shape Up Somerville: Eat Smart, Play Hard<br><br>Restaurants	21 restaurants became "Shape Up Approved" restaurants - 12% of total restaurants in Somerville (n = 171) and 28% of those that were actively recruited (n = 74) (16).	Within 6 months of signing the agreement, 10/21 restaurants fully complied with all minimum approval criteria. 11 failed to mark the healthier options, and 1 also failed to display the Shape Up Somerville seal of approval. All met the nutrition criteria.<br>Owners and managers were notified of noncompliance, follow-up visits in 4 restaurants ~3 months after initial visits found 2 restaurants had come into full compliance and 2 had not. 4/10 retailers who returned questionnaires had made changes to their menus (16).	6/10 retailers surveyed reported that customers ordered "Shape Up Approved" items from their menus at least once per week. 5/10 reported that customers asked about or mentioned Shape Up Somerville at least once per week (16).	Not reported	7/10 retailers surveyed believed that program participation had been beneficial for them. 3/10 indicated that the program had drawn a new customer base to their restaurants. 7/10 indicated that they were more aware of nutrition as a result of participating. 5/10 thought their staff were more aware of nutrition and 4/10 "strongly agreed" or "agreed" that their customers were more aware of nutrition as a result of the program (16).
¡Por Vida! Initiative<br><br>Restaurants	Not reported	44 different implementation activities included: collaboration with the local health department to implement promotions, increasing fruit and	More customers who purchased included items reported seeing the logo than not. Customer reported being able to see the	98.9% customers liked the appearance of the menus. 98.4% children liked the healthier food.	Institutions reported receiving positive attention associated with ¡Por Vida! from the media, corporate partners, and their customers.

Scheme name	Uptake and certification rates	Impact on retailer practices	Impact on customer behaviours	Customer perspectives	Retailer perspectives and commercial outcomes
		vegetable availability, and educating patrons on how to make ¡Por Vida! selections (45).	logo contributed to purchasing behaviours (46).	Consumers rated the taste of menu items as more important than their nutritional value (7, 45).	
Eat Well El Paso! (EWEP) (42)<br><br>Restaurants	For the 5 years of the program 21/30 initially recruited restaurants (4 left the scheme) were certified (70%).<br><br>Long-term retention was low; 5 restaurants continued after the 5-year program (17%).				
Corner stores					
Change4Life<br><br>Change4Life convenience store intervention	87 stores total participated. 52.7% stores used marketing equipment appropriately (1).	A number of retailers commented that the intervention prompted them to expand their FFV range.<br><br>Store uptake of Change4Life: Branded chill cabinet, shelves, stand & FFV champion (2.7%) branded chill cabinet (57% [55% used appropriately]; branded shelves 36.5% [48% used appropriately]; branded stand 31% [26% used appropriately]; FFV champion (23.0%) (1)	Sales of FFV were reported to improve following the intervention (1).	Not reported	Most retailers supported the health goals of the intervention. However, retailers' commercial interests were the key driver of participation.<br><br>Retailers' understanding of intervention aims was low (1).
Healthy HotSpot (HH) initiative<br><br>Corner stores	53 corner stores approached, 25 agreed to participate (in pilot) (28).	In addition to the required FFV, the most commonly added food type was whole grains. Low-fat dairy products were added in 10/25 stores and canned fruits or vegetables in 9/25 stores. The least popular category was lean protein (28).	Not reported	Not reported	Store owners commonly:<br>(i) Prioritized community health;<br>(ii) Engaged with the community in a positive manner;<br>(iii) Identified themselves as a health Resource for the community (13).

Scheme name	Uptake and certification rates	Impact on retailer practices	Impact on customer behaviours	Customer perspectives	Retailer perspectives and commercial outcomes
Fresh Foods Here<br><br>Corner stores	Not reported	The average quantity of healthy food items increased by an average of almost 39 items (40).	Average daily sales of healthy items increased:<br>*Pre-intervention:* 133.3<br>*During intervention:* 269.1<br>*Post intervention:* 309.5 (40).	Across periods, customers tended to place the greatest importance on eating fruits and vegetables, while eating low fat dairy foods was rated the least important (40).<br><br>The percentage of people who had heard of Fresh Foods Here increased significantly from 33.5% to 46.2% from the pre- to the post-evaluation period (40).	Average daily number of shoppers:<br>*Pre-intervention:* 642 shoppers<br>*During intervention:* 740 shoppers<br>*Post-intervention:* 694 shoppers (40).<br><br>Average daily number of transactions:<br>*Pre-intervention:* 473 transactions<br>*During intervention:* 541 transactions<br>*Post-intervention:* 527 transactions (40).
Healthy Bodegas Initiative<br><br>Corner stores	60 stores recruited to participate. 55 stores completed the study (5 lost to follow-up). Two thirds of stores advanced at least 1 healthy store level, and some advanced as many as 3 (10).	Owners made on average 4 health-promoting changes, with some making as many as 7, out of 15 criteria. Increases in the proportion of stores stocking: no added sugar canned fruit (pre: 71%; post: 96%); 4 varieties of fresh fruit (36%; 47%); refrigerated water at eye level (35%, 64%); healthy sandwich (0%, 55%), and posted signs to identify healthier items (0%; 95%) (10).	Food and beverage purchases were comparable before and after the intervention. Although the number of purchases of healthy items was low, purchases increased for some healthy foods (10).	Not reported	Of the 46 store owners who completed surveys both before and after the intervention, 78% reported that the intervention helped improve their sales of healthier foods (10).
Healthy2Go<br><br>Corner stores	Not reported	Inventory tracking showed that, on average, Healthy2Go stores increased availability of 11/21 categories of healthy products (43).<br><br>Stores in the H2G program had a 40% increase in stocking fresh produce, 20% increase in produce variety, and trends towards increasing healthy inventory (43).	There was a significant increase in the number of customer reporting they were likely to buy healthy foods at country stores (43).<br><br>Post-implementation, customers were more likely to purchase more healthy foods, than unhealthy foods, from participating corner stores (43).	At baseline 75% of the 287 surveyed community members reported a desire to eat healthier at baseline (43).<br><br>The majority of respondents stated that they would like Healthy2Go to continue (43).	100% of store owners reported planned to continue promoting healthy products. Store owners unanimously thought that Healthy2Go should expand and that the program helped their store make healthy changes (43).

Scheme name	Uptake and certification rates	Impact on retailer practices	Impact on customer behaviours	Customer perspectives	Retailer perspectives and commercial outcomes
Healthy Retail SF<br><br>Corner stores	Not reported	Stores were awarded a rating of 1 to 4 stars based on availability and promotion of healthy and unhealthy products. There was a decrease in stores with 1 or 2 stars (2013: 77%, 2017: 49%) (35).	The first 4 Tenderloin HRSF stores with full data from baseline through the first 12 months of follow-up showed a 35% increase in (healthy) produce units sold (35).	Not reported	Many of the merchants uninterested in HRSF remarked that Tenderloin residents faced challenges, including poverty, homelessness, and alcoholism and drug addiction. Some saw their role as trying to promote health (34).
FIT Store (part of Project FIT)<br><br>Corner stores	3/4 stores increased, 1/4 stores decreased in NEM-S score (39).	3 out of 4 stores increased availability of healthy products. Availability of fresh vegetables declined slightly or remained constant across all stores.<br>All stores sold 100% whole wheat bread and low-sugar/high-fibre cereal.<br>2 stores' price points decreased, 1 store's increased, and 1 store's stayed the same (39).	Respondents self-reported increased purchases compared to 1 year ago of grain food (40.8%); protein (38.0%); low-fat dairy (40%); fruit/vegetable (46.3%) (39).	Post-intervention, significantly more customer survey respondents had heard about Project FIT.<br>80.9% of the respondents believed that the store made a variety of healthy foods available and 64.9% felt that the store reduced the prices of healthy foods (39).	Not reported
United States Department of Agriculture's Special Supplemental Nutrition Program for Women, Infants, and Children (WIC)<br><br>Supplemental Nutrition Assistance Program (SNAP)<br><br>Corner stores	Not reported	Upgraded stores had significantly higher Short-form Corner store Audit Tool (SCAT) scores compared to non-upgraded stores. Upgraded versus non-upgraded store status was not associated with NEMS-CS-Availability scores (12).	Not reported	Not reported	Not reported
Schools and childcare settings					

Scheme name	Uptake and certification rates	Impact on retailer practices	Impact on customer behaviours	Customer perspectives	Retailer perspectives and commercial outcomes
ABC Grow Healthy nutrition standards<br><br>Early care and education (ECE) centres	261 centres participated.	Compared with non-participating centres, more participating centres reported having a written nutrition policy (95.8% v 83.3%), requiring staff to attend nutrition training more than once per year (46.6% v 20.9%), and serving healthy foods or non-food treats to celebrate holidays or events (80.3% v 59.4%). Fewer participating centres reported serving children juice drinks containing <100% fruit juice (2.7% vs. 23.5%) (48).	Not reported	Not reported	Not reported
Start Right - Eat Right (SRER)<br><br>Long Day Care Centres	*South Australia:* 44/50 (88%) centres expressed interest. 25/44 (57%) certification rate.<br><br>*Western Australia:* 134/418 centres expressed interest. 75 awarded full completion.	*South Australia:* Substantial menu changes across centres, increased serving of fruits and vegetables, meats and alternatives, and dairy products (5, 6, 33).<br><br>*Western Australia:* Forty-six (90%) of coordinators reported making changes to menus. 39/46 (81%) centres increased milk, 8 (17%) increased meat, and 6 (12%) increased the variety of food served.<br>18/25 centres (out of 25 that registered) reported making changes as a result of taking part in the award scheme—8 made changes to menus; 5 reported increased awareness of food, nutrition, hygiene, and health issues; and 5 reported changes or updates to nutrition policies (41).	*South Australia:*<br>Scheme participation resulted in increased mean daily intake amongst children in core foods (baseline: 0.1 serves; post-implementation: 0.5 serves) and energy intake (baseline: 1630kJ, post-implementation: 1791kJ) and improvements in intakes of eleven out of the 19 nutrients evaluated were observed (proportion of intake as saturated fat decreased by 1 percentage point, proportion of children meeting fibre, calcium and sodium benchmarks increased by 10, 9, 21 percentage points, respectively) (6).	Not reported	*South Australia:* Centres reported changes in staff knowledge and an ability to self-review and develop menus that complied with nutritional benchmarks (5, 33).<br><br>*Western Australia:* 47/51 centres that were registered in the scheme but not applied for the award thought the short course was relevant to the coordinator and cook and that it increased knowledge (41).<br><br>16/21 centres that had received the award were satisfied with the award. 17 said that it had benefited the centre by improving knowledge in nutrition and food service, preparing for accreditation, reassuring parents, and improving cooperation between the cook and coordinator (41).

Scheme name	Uptake and certification rates	Impact on retailer practices	Impact on customer behaviours	Customer perspectives	Retailer perspectives and commercial outcomes
USDA Child and Adult Care Food Program (CACFP)<br><br>Childcare centres	181 of 636 centres that serve food were CACFP centres	CACFP centres were considerably more likely than non-CACFP centres to provide breakfast and lunch. CACFP centres reported more engagement in recommended feeding and nutrition practices than non-CACFP centres, including:<br>-Serving whole fruit at lunch (100% v 94%)<br>-Serving whole grain at snack time (95% v 76%)<br>-Serving low fat milk at snack time (93% v 67%)<br>-Use of family style dining (86% to 54%)<br>-Provide nutrition training for their staff (91% v 55%)<br>-Prohibiting food and beverages that staff can bring from home to consume in view of children (75% v 56%)<br>-CACFP-participating centres reported significantly better adherence to the CACFP nutrition standards (2).	Not reported	Not reported	53% of CACFP respondents thought that having centre-provided meals/snacks was an important factor in parental decisions to enrol. Only 20% of non-CACFP centres thought it was an important enrolment consideration among parents (2).<br><br>CACFP-participating retailers felt that participation resulted in better dietary quality of served foods, higher food costs, and increased food waste (3).<br><br>Federal funds are received for meal reimbursements to CACFP-compliant sites. The state of Connecticut was estimated to missed out on USD30,656,804 in federal funds in over the 2019−2020 financial year (3).
Hospitals					
The Healthy Hospital Food Initiative<br><br>Hospitals	Participating hospitals represented more than 60% of all acute-care facilities in NYC (36).<br><br>All 16 public hospitals had implemented the standards for patient meals, beverage vending, and food vending before the HHFI. By September 2014, 2 (12%) of the 16 public hospitals had	Between baseline and the end of the study, 54% of hospitals implemented the breakfast pastry, 54% of hospitals implemented dessert standards, 64% removed unhealthy foods and beverages from the entrance and checkout of the cafeteria, 61% offered proportionally priced half-size sandwiches, 39% offered only soups that met the sodium limit and labelled all menu items with calories, 39%	Not reported	Not reported	Not reported

Scheme name	Uptake and certification rates	Impact on retailer practices	Impact on customer behaviours	Customer perspectives	Retailer perspectives and commercial outcomes
	implemented the Standards for Cafeterias/Cafés.<br>At the end of the study, 17 (71%) private hospitals had implemented the Standards for Patient Meals, 14 (58%) had implemented the Standards for Beverage Vending Machines, 12 (50%) had implemented the Standards for Food Vending Machines, and 16 (67%) had implemented the Standards for Cafeterias/Cafés (36).<br><br>All 16 public hospitals began at silver star status, and 2 hospitals reached gold. 9 private hospitals (38%) reached gold, 7 (29%) reached silver, 3 (13%) reached bronze, and 5 (21%) joined the HHFI but did not implement any standards (36).	reduced sugary drinks to 25% of all beverages, 43% reduced portion sizes of sugary drinks to 16 ounces or smaller, 11% completely removed the deep-fat fryer, 21% met the criteria for sodium in sandwiches, salads, and entrees, 29% met the criteria for pre-packaged snacks (36).			
Workplaces					
Eat Smart! Workplace Cafeteria Program<br><br>Workplaces	Not reported	At the single site, promotional materials were used, and healthier menu options and bundle deals were available. Condiments were only available on request (11).	87% of customers reported that they had purchased something in the cafeteria. The most frequently reported change in eating habits was eating more whole-grain (42% of customers) (11).	Customers reported several program benefits:<br>(i) Increased knowledge about healthy eating<br>(ii) Convenience<br>(iii) Physical benefits (increased energy, weight loss)<br>(iv) Improved morale (11).	Not reported
Multiple settings					

Scheme name	Uptake and certification rates	Impact on retailer practices	Impact on customer behaviours	Customer perspectives	Retailer perspectives and commercial outcomes
Waupaca Eating Smart (WES)<br><br>Restaurants and supermarkets	7/9 (78%) restaurants and 2/3 (67%) food stores approached for participation signed written agreements to participate in WES. All implemented some WES activities (32).	*Restaurants:*<br>Pre-intervention, only 1 of 7 restaurants had ≥3meals that met WES nutrition criteria. Post-intervention, 38 meals were labelled and promoted to restaurant customers.<br><br>*Supermarkets:*<br>At the mid-intervention point, both supermarkets implemented 9 of 12 WES strategies. Post-intervention, staff at one supermarket no longer actively recommended WES items (17).	About 15.5% of surveyed customers reported purchasing foods promoted by WES signs or materials from restaurants and stores (32).	*Restaurants:*<br>51.0% of customers reported they had heard of WES, 60.9% recognized the WES logo, and 36.9% had noticed the WES logo. On a 0 to 4 scale, customers who noticed WES materials reported that, on average, ease of understanding WES materials was 3.1, level of appeal was 2.1, and helpfulness in deciding what to order was 0.9 (32).<br><br>*Stores:*<br>50.5% of customers reported they had heard of WES, 59.1% recognized the WES logo, and 50.0% had noticed the WES logo. On a 0 to 4 scale, customers who noticed WES materials reported that, on average, ease of understanding WES materials was 3.0, level of appeal was 2.3, and helpfulness in deciding what to order was 1.2 (32).	On a 0-4 scale (0=not likely, 4=very likely), the average likelihood of continuing implementing WES at the end of the 10-month evaluation period was 2.86 for restaurant managers and 3.5 for store managers. On a 0 to 4 scale, the average impact on business was 3.0 and 2.0 for restaurant and store managers, respectively. The average level of satisfaction with WES was 3.14 for restaurant managers and 3.0 for store managers (17, 32).
Food for Life Partnership (FFLP)<br><br>Schools, universities, care homes, hospitals.	Each of 3 hospitals reached certification and received a 'catering mark' (21, 22).<br><br>Schools had the opportunity to reach different levels of accreditation. 7 schools had achieved the Gold Mark, 53 the Silver Mark and 132 the Bronze Mark. A further 2797 schools	Hospitals reported better quality patient food being served; healthier choices for staff and visitor dining; nutritional support for patients at discharge; better co-ordination of food related activity, and; improved understanding of the role food plays in patient care and recovery (21, 22).<br><br>Amongst 38 schools, use of local suppliers in school procurement rose by 73%.	Two years of the FFLP flagship programme, school meal take up for primary schools rose from 45.4% to 49.2% and school meal take up for secondary schools rose from 50.3% to 56.0% (38).	Not reported	One view strongly expressed was that a visit to a hospital should be an opportunity to model an exemplar food offering, for staff to be role models, and to provide knowledge about how to make those healthier choices (21, 22).

Scheme name	Uptake and certification rates	Impact on retailer practices	Impact on customer behaviours	Customer perspectives	Retailer perspectives and commercial outcomes
	were registered but had no current award (38).	Significant investment was made in the kitchen environment by FFLP schools. 24% of schools reported using sustainably sourced ingredients on a regular basis (38).			
Heartbeat award (HBA) scheme<br><br>Workplaces, restaurants, cafés, schools, residential homes, sports centres	25 premises in Bedfordshire implement the Award (44).<br><br>4/6 premises in Leicestershire achieved the award (26).	Many premises (across multiple localities) promoted healthy choices as their only HBA activity. Limited evidence of changes to menus.	Limited evidence.<br><br>In one case, retailers reported increases in purchasing of several healthier food items since receiving the HBA. Work-place caterers reported most changes in uptake (25).<br><br>In another study, in HBA workplaces, according to employee-completed food frequency questionnaires, there was an increase in consumption of fruit; reduction in consumption of fried foods and sweet puddings; greater use of low fat milks (27).<br><br>A third study saw both HBA and non-HBA premises increase healthy food sales (47).	Customer awareness and understanding of the HBA was consistently low. Customer use of healthier product labels was moderate (24, 44).	Commercial advantages and consumers demand were seen as positive reasons to participate (47).
Heartbeat Award (HBA) administered by Heartbeat Wales (HBW)<br><br>Workplaces, restaurants, cafés, schools, residential homes, sports centres	Uptake of 17 (4 pilot and additional 13 authorities taking up HBA) of 22 total local authorities (77%) (37).	67% of establishments promoted the award to their clients (37).	Outcomes were given a score by establishments out of 5. Scores indicate the ability of the scheme to meet stated objective.<br><br>"Increasing the proportion of customers choosing healthy food" was scored 3.57 (37).	Not reported	Outcomes were given a score by establishments out of 5. Mean scores were:<br>"Promoting a good relationship with the Environmental Health Department" (4.54); “Increasing publicity” (3.23); “Increasing customer numbers” (2.96); “Increasing turnover” (2.69) (37).

Table S8: Accreditation scheme impact counting for included studies

First author surname, Year of publication	Accreditation scheme name	Study quality	Impact on retailer practices	Impact on customer behaviours	Customer perspectives	Retailer perspectives and commercial outcomes
Restaurants						
Gase, 2015 (18)	Choose Health LA Restaurants	Medium	+			
Gase, 2016 (34)	Choose Health LA Restaurants	Medium			+	
Macaskill, 2003 (33)	Eat Smart! Ontario's Healthy Restaurant Program	Low	-			+
Dwyer, 2004 (84)	Eat Smart! Ontario's Healthy Restaurant Program	Medium				
Redelfs, 2021 (60)	Eat Well El Paso!	Medium				
Fitzpatrick, 1997 (32)	Fresh Choice	High			+	
Bagwell, 2013 (16)	Healthier Catering Commitment (HCC)	Medium	-			
Boelsen-Robinson, 2020 (62)	Healthier Catering Commitment (HCC)	High				
Dwivedi, 1999 (30)	Heart Smart Heart Beat Restaurant Program	Low	-	-		+
Green, 1993 (31)	Heart Smart Heart Beat Restaurant Program	Low	+	-	-	
Biediger-Friedman, 2014 (26)	¡Por Vida! menu labelling initiative	Low			+	+
Sosa, 2014 (27)	¡Por Vida! (Workplaces)	Medium	+		+	+
Sosa, 2014 (28)	¡Por Vida! menu labelling initiative	High		+		
Economos, 2009 (63)	Shape Up Somerville: Eat Smart, Play Hard	High	-	+		+
Brown, 2017 (29)	Savvy Diner	Low				
Corner stores						
Adams, 2012 (42)	Change4Life Convenience Store intervention	High	+	+		+
Paluta, 2019 (39)	Fresh Foods Here	High	+	+	+	+
Lynch, 2021 (61)	Good Food Corner Stores	High				
Dannefer, 2012 (38)	Healthy Bodegas Initiative	Medium	+	+		+
Dombrowski, 2019 (41)	Healthy HotSpot	Medium				+
Jaskiewicz, 2013 (40)	Healthy HotSpot	High	+			
McDaniel, 2018 (36)	Healthy Retail SF	High				
Minkler, 2019 (35)	Healthy Retail SF	Low	+	+		
Rushakoff, 2017 (37)	Healthy2Go	Medium	+	+	+	+
Paek, 2014 (68)	Project FIT	High	+	+	+	

First author surname, Year of publication	Accreditation scheme name	Study quality	Impact on retailer practices	Impact on customer behaviours	Customer perspectives	Retailer perspectives and commercial outcomes
DeWeese, 2016 (59)	United States Department of Agriculture's Special Supplemental Nutrition Program for Women, Infants, and Children (WIC) Supplemental Nutrition Assistance Program (SNAP)	Medium	+			
Schools and childcare settings						
Zaltz, 2018 (72)	ABC Grow Healthy nutrition standards	High	+			
Bell, 2012 (43)	Start Right - Eat Right (SRER)	High	+			+
Bell, 2015 (46)	Start Right - Eat Right (SRER)	High	+	+		
Matwiejczyk, 2007 (44)	Start Right - Eat Right (SRER)	Low	+			+
Pollard, 2001 (45)	Start Right - Eat Right (SRER)	Medium	+			+
Andreyeva, 2018 (47)	USDA Child and Adult Care Food Program (CACFP)	High	+			
Andreyeva, 2022 (70)	USDA Child and Adult Care Food Program (CACFP)	High				+
Hospitals						
Moran, 2016 (48)	The Healthy Hospital Food Initiative	High	+			
Workplaces						
Dawson, 2006 (49)	Eat Smart! Workplace Cafeteria Program	Medium	+	+	+	
Multiple settings						
Gray, 2015 (53)	Food for Life Partnership	Low	+			
Gray, 2017 (52)	Food for Life Partnership	High	+			
Orme, 2011 (54)	Food for Life Partnership	High	+	+		
Holdsworth, 1997 (55)	Heartbeat Award (HBA)	Medium	-		-	
Holdsworth, 1999 (57)	Heartbeat Award (HBA)	High	-	+		
Holdsworth, 2000 (66)	Heartbeat Award (HBA)	High	-			
Holdsworth, 2004 (64)	Heartbeat Award (HBA)	Medium	-	+		
MacAuslan, 1995 (67)	Heartbeat Award (HBA)	Low	-			
Snowdon, 1998 (56)	Heartbeat Award (HBA)	Low	-		-	
Warm, 1997 (85)	Heartbeat Award (HBA)	Low	-	-		
Murphy, 1994 (58)	Heartbeat Award Wales (HBA Wales)	Low	+	+		+
Escaron, 2016 (51)	Waupaca Eating Smart	Low	+			+
Martinez-Donate, 2015 (50)	Waupaca Eating Smart	Medium		-	+	+

References

1. Adams J, Halligan J, Burges Watson D, et al. The Change4Life convenience store programme to increase retail access to fresh fruit and vegetables: a mixed methods process evaluation. PloS one. 2012;7(6):e39431.

2. Andreyeva T, Henderson KE. Center-Reported Adherence to Nutrition Standards of the Child and Adult Care Food Program. Childhood obesity (Print). 2018;14(6):421-8.

3. Andreyeva T, Sun X, Cannon M, et al. The Child and Adult Care Food Program: Barriers to Participation and Financial Implications of Underuse. Journal of nutrition education and behavior. 2022;54(4):327-34.

4. Bagwell S. Healthier catering initiatives in London, UK: an effective tool for encouraging healthier consumption behaviour? Critical Public Health. 2014;24(1):35-46.

5. Bell L. South Australian Long Day Care Centres engaged with a nutrition incentive award scheme show consistency with mealtime practice guidelines. Nutrition &amp Dietetics. 2012;69:130-6.

6. Bell LK, Hendrie GA, Hartley J, et al. Impact of a nutrition award scheme on the food and nutrient intakes of 2- to 4-year-olds attending long day care. Public Health Nutr. 2015;18(14):2634-42.

7. Biediger-Friedman L, Sosa E, Shields K, et al. A Voluntary Approach to Improve Menu Options in Restaurants Through a Local Collab- orative Partnership. The Journal of the Egyptian Public Health Association. 2014;66:11-4.

8. Boelsen-Robinson T, Peeters A, Thow AM, et al. Barriers and facilitators to implementing a healthier food outlet initiative: perspectives from local governments. Public health nutrition. 2020:1-13.

9. Brown T, Vanderlinden L, Birks A, et al. Bringing Menu Labelling to Independent Restaurants: Findings from a Voluntary Pilot Project in Toronto. Canadian journal of dietetic practice and research : a publication of Dietitians of Canada = Revue canadienne de la pratique et de la recherche en dietetique : une publication des Dietetistes du Canada. 2017;78(4):177-81.

10. Dannefer R, Williams DA, Baronberg S, et al. Healthy Bodegas: Increasing and Promoting Healthy Foods at Corner Stores in New York City. American Journal of Public Health. 2012;102(10):e27-e31.

11. Dawson J, Dwyer JJM, Evers S, et al. Eat smart! Workplace Cafeteria Program evaluation of the nutrition component. Canadian journal of dietetic practice and research : a publication of Dietitians of Canada = Revue canadienne de la pratique et de la recherche en dietetique : une publication des Dietetistes du Canada. 2006;67(2):85-90.

12. DeWeese RS, Todd M, Karpyn A, et al. Healthy store programs and the Special Supplemental Nutrition Program for Women, Infants, and Children (WIC), but not the Supplemental Nutrition Assistance Program (SNAP), are associated with corner store healthfulness. Preventive medicine reports. 2016;4:256-61.

13. Dombrowski RD, Kelley MA. Corner Store Owners as Health Promotion Agents in Low-Income Communities. Health Education & Behavior. 2019;46(6):905-15.

14. Dwivedi G, Harvey J. Evaluation of the Heart Smart(tm) Heart Beat Restaurant Program. Can J Diet Pract Res. 1999;60(3):159.

15. Dwyer JJ, Macaskill LA, Uetrecht CL, et al. Eat Smart! Ontario's Healthy Restaurant Program: focus groups with non-participating restaurant operators. Canadian journal of dietetic practice and research : a publication of Dietitians of Canada = Revue canadienne de la pratique et de la recherche en diététique : une publication des Diététistes du Canada. 2004;65(1):6-9.

16. Economos CD, Folta SC, Goldberg J, et al. A community-based restaurant initiative to increase availability of healthy menu options in Somerville, Massachusetts: Shape Up Somerville. Preventing chronic disease. 2009;6(3):A102.

17. Escaron AL, Martinez-Donate AP, Riggall AJ, et al. Developing and implementing "Waupaca Eating Smart": a restaurant and supermarket intervention to promote healthy eating through changes in the food environment. Health Promotion Practice. 2016;17(2):265-77.

18. Fitzpatrick MP, Chapman GE, Barr SI. Lower-fat menu items in restaurants satisfy customers. Journal of the American Dietetic Association. 1997;97(5):510-4.

19. Gase LN, Kaur M, Dunning L, et al. What menu changes do restaurants make after joining a voluntary restaurant recognition program? Appetite. 2015;89:131-5.
20. Gase LN, Montes C, Robles B, et al. Media outlet and consumer reactions to promotional activities of the Choose Health LA Restaurants program in Los Angeles County. Journal of Public Health Management and Practice. 2016;22(3):231-44.
21. Gray S, Means R, Orme J, et al. Improving Hospital Food: evaluating the impact of the UK Food for Life Partnership. European Journal of Public Health. 2015;25(suppl_3):ckv176.018.
22. Gray S, Orme J, Pitt H, et al. Food for Life: evaluation of the impact of the Hospital Food Programme in England using a case study approach. JRSM open. 2017;8(10):2054270417712703.
23. Green KL, Steer SL, Maluk RE, et al. Evaluation of the Heart Smart Restaurant Program in Saskatoon and Regina, Saskatchewan. Canadian journal of public health = Revue canadienne de sante publique. 1993;84(6):399-402.
24. Holdsworth M, Haslam C, Raymond NT, et al. Evaluation of customers' perspectives on the heartbeat award scheme in public eating places. Journal of Nutrition Education. 1997;29(5):231-6.
25. Holdsworth M, Haslam C, Raymond NT. An assessment of compliance with nutrition criteria and food purchasing trends in Heartbeat Award premises. Journal of Human Nutrition and Dietetics. 1999;12(4):327-35.
26. Holdsworth M, Haslam C, Raymond NT. Does the heartbeat award scheme change employees' dietary attitudes and knowledge? Appetite. 2000;35(2):179-88.
27. Holdsworth M, Raymond NT, Haslam C. Does the Heartbeat Award scheme in England result in change in dietary behaviour in the workplace? Health Promotion International. 2004;19(2):197-204.
28. Jaskiewicz L, Dombrowski RD, Drummond HM, et al. Partnering with community institutions to increase access to healthful foods across municipalities. Preventing chronic disease. 2013;10:E167.
29. Lynch M, Graham M, Taylor K, et al. Corner store retailers' perspectives on a discontinued Healthy Corner Store Initiative. International quarterly of community health education. 2021:272684X211004930.
30. Macaskill LA, Dwyer JJM, Uetrecht CL, et al. Eat Smart! Ontario's Healthy Restaurant Program: a survey of participating restaurant operators. Canadian journal of dietetic practice and research : a publication of Dietitians of Canada = Revue canadienne de la pratique et de la recherche en dietetique : une publication des Dietetistes du Canada. 2003;64(4):202-7.
31. MacAuslan EM. The heartbeat award: Is attainment of the current criteria by eat-in sandwich bars practicable? Health and Hygiene. 1995;16:163-7.
32. Martínez-Donate AP, Riggall AJ, Meinen AM, et al. Evaluation of a pilot healthy eating intervention in restaurants and food stores of a rural community: a randomized community trial. BMC Public Health. 2015;15:136.
33. Matwiejczyk L, McWhinnie JA, Colmer K. An evaluation of a nutrition intervention at childcare centres in South Australia. Health Promot J Austr. 2007;18(2):159-62.
34. McDaniel PA, Minkler M, Juachon L, et al. Merchant attitudes toward a healthy food retailer incentive program in a low-income San Francisco neighborhood. Int Q Community Health Educ. 2018;38(4):207-15.
35. Minkler M, Estrada J, Dyer S, et al. Healthy Retail as a Strategy for Improving Food Security and the Built Environment in San Francisco. American journal of public health. 2019;109(S2):S137-S40.
36. Moran A, Krepp EM, Johnson Curtis C, et al. An Intervention to Increase Availability of Healthy Foods and Beverages in New York City Hospitals: The Healthy Hospital Food Initiative, 2010-2014. Preventing chronic disease. 2016;13:E77.
37. Murphy S, Powell C, Smith C. A formative evaluation of the Welsh Heartbeat Award Scheme. Nutrition and Health. 1994;9(4):317-27.
38. Orme J, Jones M, Kimberlee R, et al., editors. Food for Life Partnership Evaluation Full Report2011.
39. Paek H, Oh H, Jung Y, et al. Assessment of a healthy corner store program (FIT store) in low-income, urban, and ethnically diverse neighborhoods in Michigan. Family and Community Health. 2014;37(1):86-99.
40. Paluta L, Kaiser ML, Huber-Krum S, et al. Evaluating the impact of a healthy corner store initiative on food access domains. Evaluation & Program Planning. 2019;73:24-32.

41. Pollard C, Lewis J, Miller M. Start right-eat right award scheme: implementing food and nutrition policy in child care centers. Health Educ Behav. 2001;28(3):320-30.
42. Redelfs AH, Leos JD, Mata H, et al. Eat Well El Paso!: Lessons Learned From a Community-Level Restaurant Initiative to Increase Availability of Healthy Options While Celebrating Local Cuisine. American journal of health promotion : AJHP. 2021;35(6):841-4.
43. Rushakoff JA, Zoughbie DE, Bui N, et al. Evaluation of Healthy2Go: A country store transformation project to improve the food environment and consumer choices in Appalachian Kentucky. Preventive medicine reports. 2017;7:187-92.
44. Snowdon W. Bedfordshire Heartbeat Award: Research into caterer and customer perceptions of this award. International Journal of Health Promotion and Education. 1998;36(2):60-3.
45. Sosa ET, Biediger-Friedman L, Shields K, et al. Increasing healthy choices in the workplace using ¡Por Vida! Health Behavior and Policy Review. 2014;1(3):238-46.
46. Sosa ET, Biediger-Friedman L, Banda M. Associations between a voluntary restaurant menu designation initiative and patron purchasing behavior. Health Promotion Practice. 2014;15(2):281-7.
47. Warm DL, Rushmere AE, Margetts BM, et al. The Heartbeat Award Scheme: an evaluation of catering practices. Journal of Human Nutrition and Dietetics. 1997;10(3):171-9.
48. Zaltz DA, Pate RR, O'Neill JR, et al. Barriers and facilitators to compliance with a state healthy eating policy in Early Care and Education Centers. Childhood obesity (Print). 2018;14(6):349-57.
